# Supplementary material for: Chemical Composition, Antioxidant and Anti-Inflammatory Activity of Shiitake Mushrooms (Lentinus edodes)
Source: J Fungi (Basel). 2024 Aug 5;10(8):552. doi: 10.3390/jof10080552 (PMC11355609; doi:10.3390/jof10080552)
Supplement: Supplementary file 1 [file jof-10-00552-s001.zip › jof-3115477-supplementary.pdf]

Supplemental Information

Supporting Figures

Figure S1. The ^{1}H-NMR (600 MH*z*) and ^{13}C-NMR (150 MH*z*) spectrums of compound **1** in DMSO-d_6.

Figure S2. The HR-ESI-MS, UV and IR spectrums of compound **1**.

Figure S3. The CD, HSQC (600 MH*z*) and HMBC (600 MH*z*) spectrums of compound **1**.

Figure S4. The NOESY (600 MH*z*) spectrum of compound **1**.

Figure S5. The ^{1}H-NMR (600 MH*z*) and ^{13}C-NMR (150 MH*z*) spectrums of compound **2** in DMSO-d_6.

Figure S6. The HR-ESI-MS, UV and IR spectrums of compound **2**.

Figure S7. The CD, HSQC (600 MH*z*) and HMBC (600 MH*z*) spectrums of compound **2**.

Figure S8. The ^{1}H-NMR (600 MH*z*) and ^{13}C-NMR (150 MH*z*) spectrums of compound **3** in DMSO-d_6.

Figure S9. The HR-ESI-MS, UV and IR spectrums of compound **3**.

Figure S10. The HSQC, HMBC and ^{1}H-^{1}H COSY spectrums (600 MH*z*) of compound **3**.

Figure S11. The ^{1}H-NMR (400 MH*z*) and ^{13}C-NMR (100 MH*z*) spectrums of compound **4** in DMSO-d_6.

Figure S12. The HR-ESI-MS, UV and IR spectrums of compound **4**.

Figure S13. The CD spectrum of compound **4**.

Figure S14. The ^{1}H-NMR (400 MH*z*) and ^{13}C-NMR (100 MH*z*) spectrums of compound **5** in DMSO-d_6.

Figure S15. The HR-ESI-MS, UV and IR spectrums of compound **5**.

Figure S16. The CD spectrum of compound **5**.

Figure S17. The HSQC and HMBC spectrums (400 MH*z*) of compound **5**.

Figure S18. The ^{1}H-NMR (400 MH*z*) spectrum of compound **6** in DMSO-d_6.

Figure S19. The ^{1}H-NMR (400 MH*z*) and ^{13}C-NMR (100 MH*z*) spectrums of compound **7** in DMSO-d_6.

Figure S20. The ^{1}H-NMR (400 MH*z*) and ^{13}C-NMR (100 MH*z*) spectrums of compound **8** in DMSO-d_6.

Figure S21. The ^{1}H-NMR (400 MH*z*) and ^{13}C-NMR (100 MH*z*) spectrums of compound **9** in DMSO-d_6.

Figure S22. The ^{1}H-NMR (400 MH*z*) and ^{13}C-NMR (100 MH*z*) spectrums of compound **10** in DMSO-d_6.

Figure S23. The ^{1}H-NMR (400 MH*z*) and ^{13}C-NMR (100 MH*z*) spectrums of compound **11** in DMSO-d_6.

Figure S24. The ^{1}H-NMR (400 MH*z*) and ^{13}C-NMR (100 MH*z*) spectrums of compound **12** in DMSO-d_6.

Figure S25. The ^{1}H-NMR (400 MH*z*) and ^{13}C-NMR (100 MH*z*) spectrums of compound **13** in DMSO-d_6.

Figure S26. The ^{1}H-NMR (400 MH*z*) spectrum of compound **14** in DMSO-d_6.

Figure S27. The ^{1}H-NMR (400 MH*z*) spectrum of compound **15** in DMSO-d_6.

Figure S28. The ^{1}H-NMR (400 MH*z*) spectrum of compound **16** in DMSO-d_6.

Figure S29. The ^{1}H-NMR (400 MH*z*) spectrum of compound **17** in DMSO-d_6.

Figure S30. The ^{1}H-NMR (400 MH*z*) and ^{13}C-NMR (100 MH*z*) spectrums of compound **18** in DMSO-d_6.

Figure S31. The ^{1}H-NMR (400 MH*z*) spectrum of compound **19** in DMSO-d_6.

Figure S32. The ^{1}H-NMR (400 MH*z*) spectrum of compound **20** in DMSO-d_6.

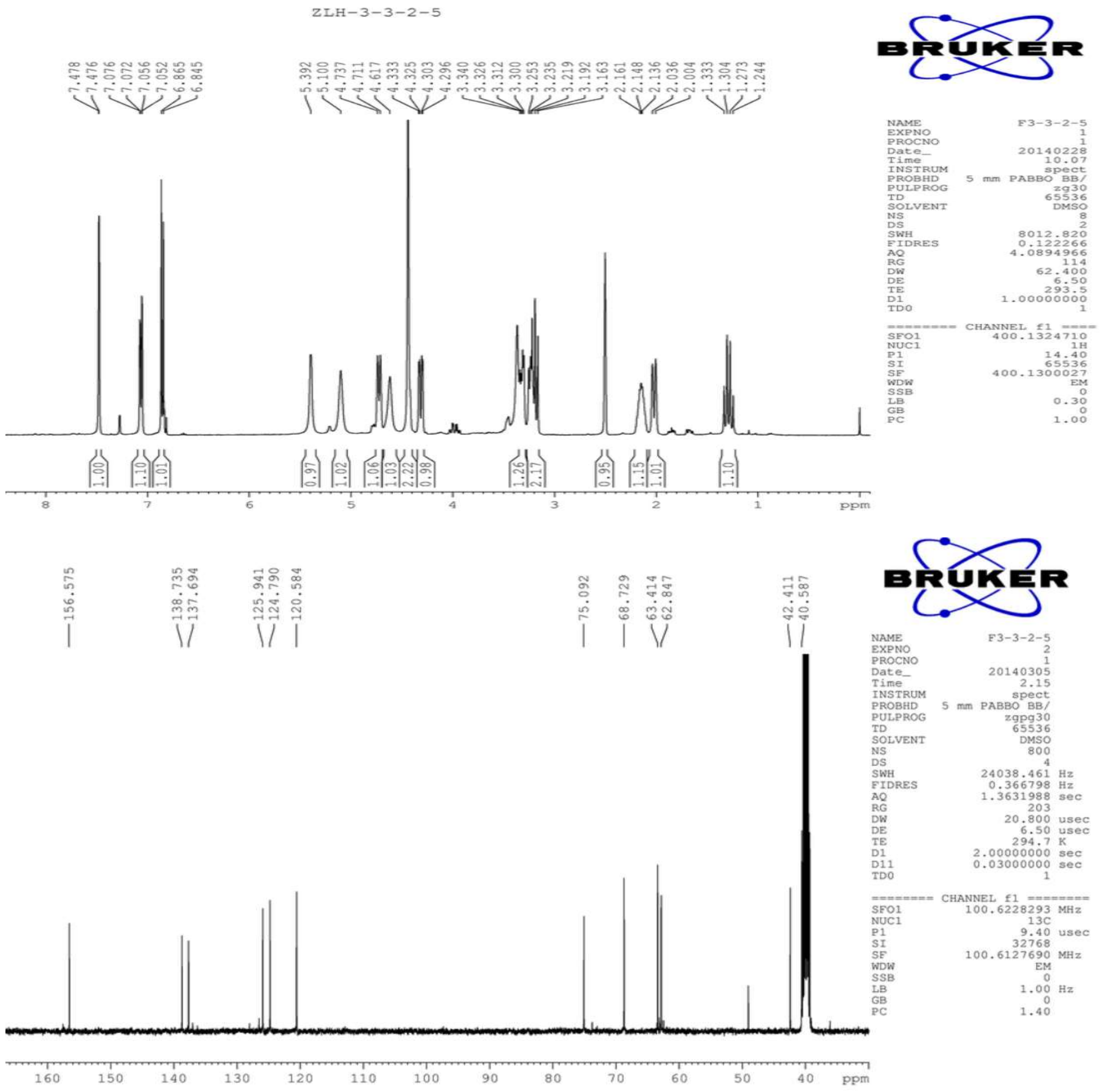

Figure S1. The ^{1}H-NMR (600 MH*z*) and ^{13}C-NMR (150 MH*z*) spectrums of compound **1** in DMSO-d_6.

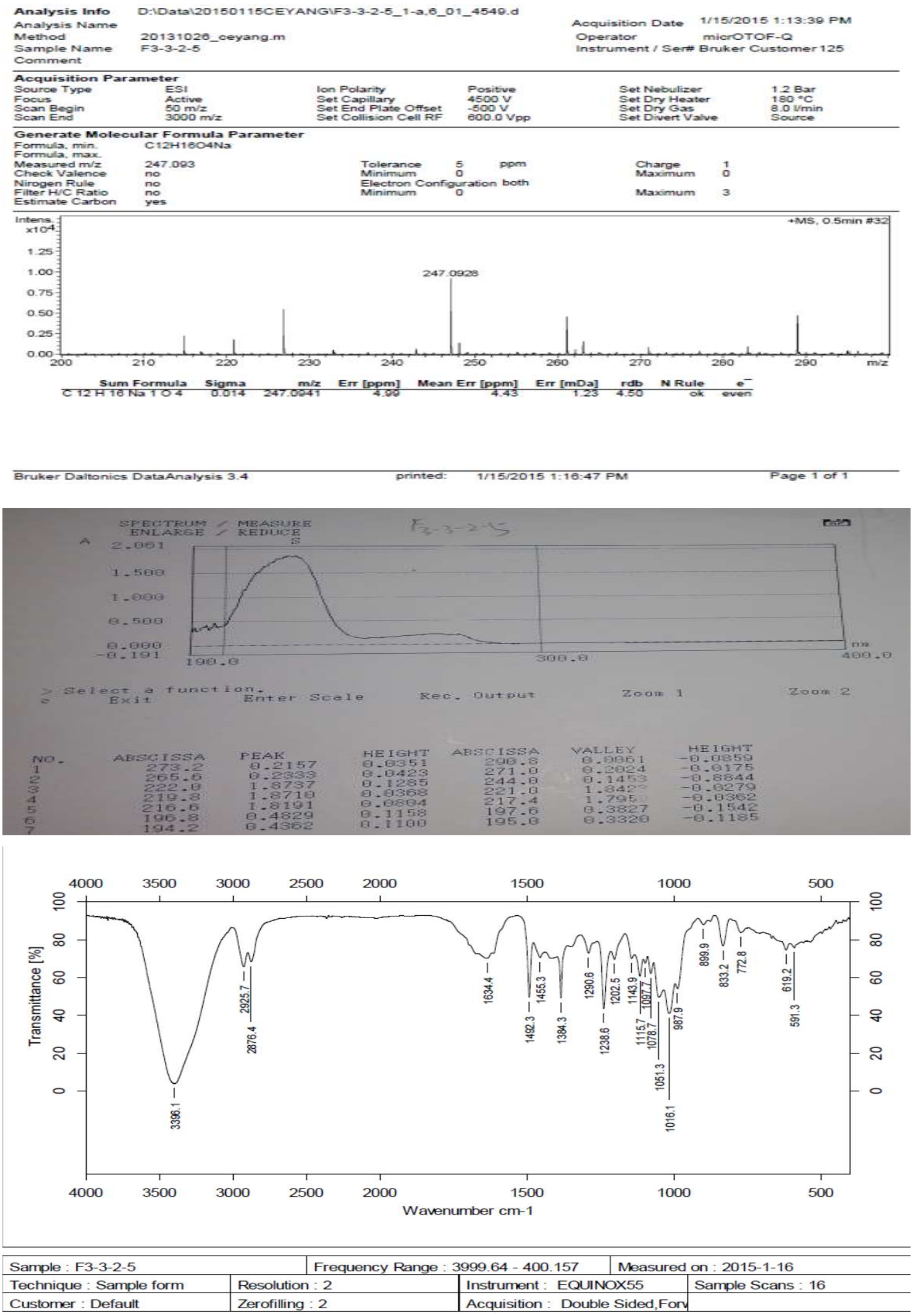

Figure S2. The HR-ESI-MS, UV and IR spectrums of compound **1**.

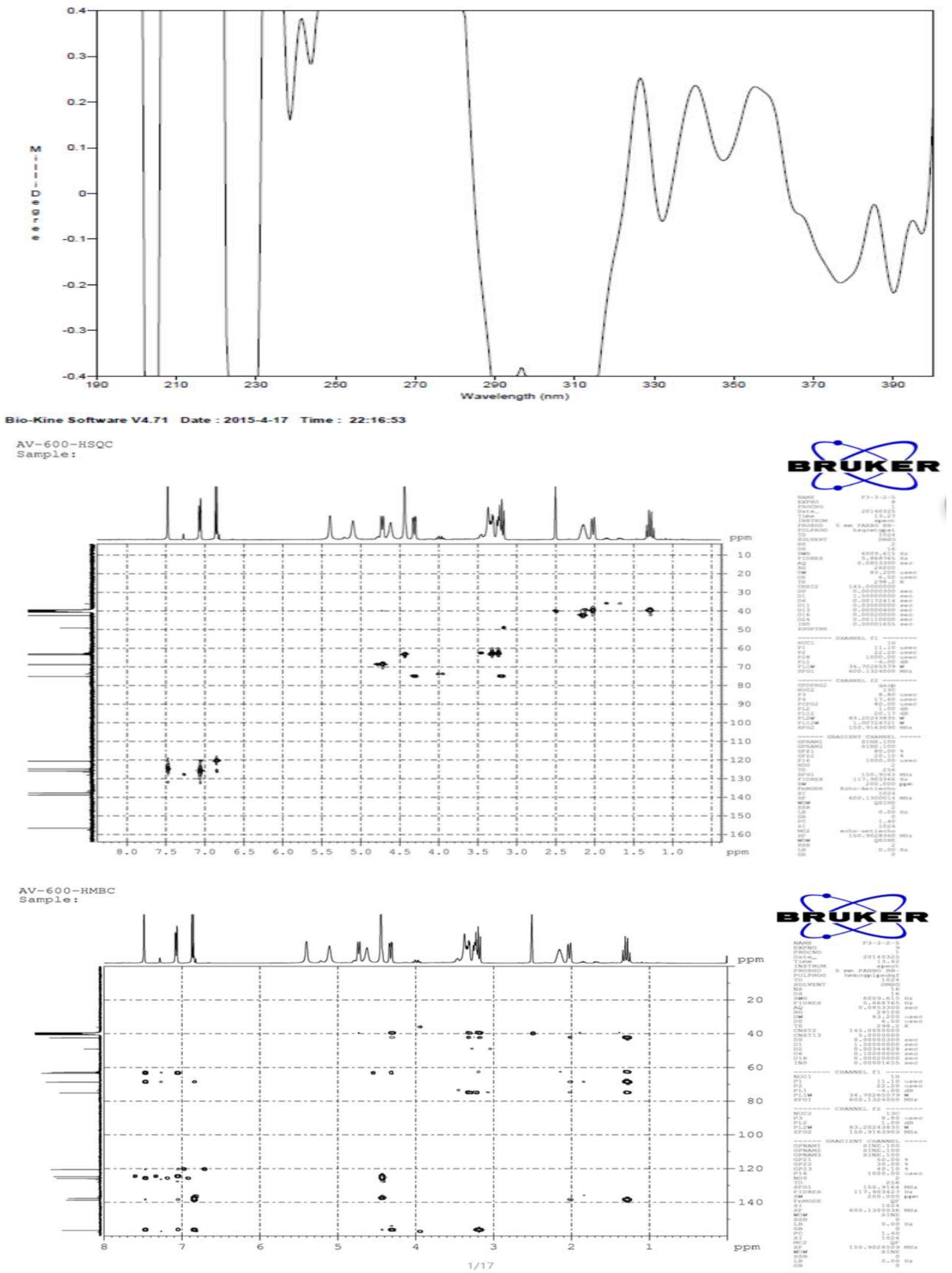

Figure S3. The CD, HSQC (600 MH*z*) and HMBC (600 MH*z*) spectrums of compound **1**.

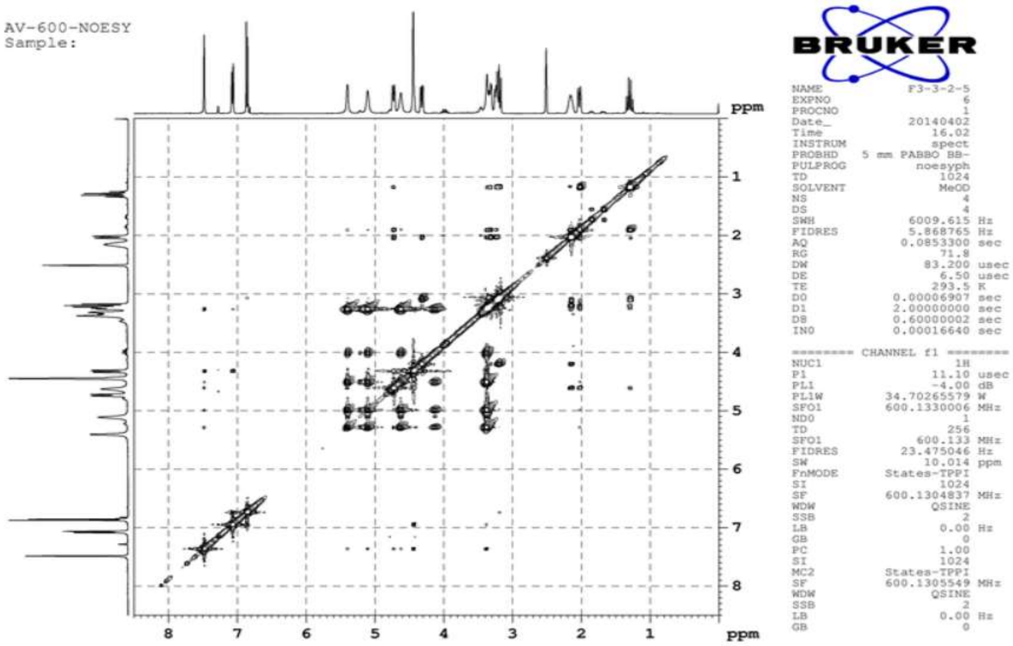

Figure S4. The NOESY (600 MH*z*) spectrum of compound **1**.

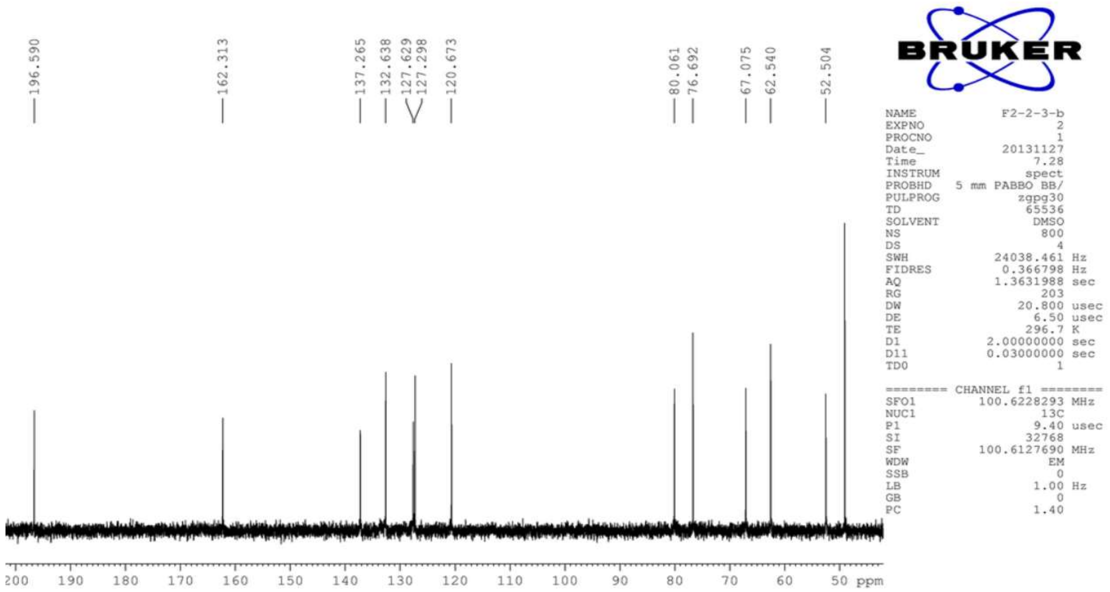

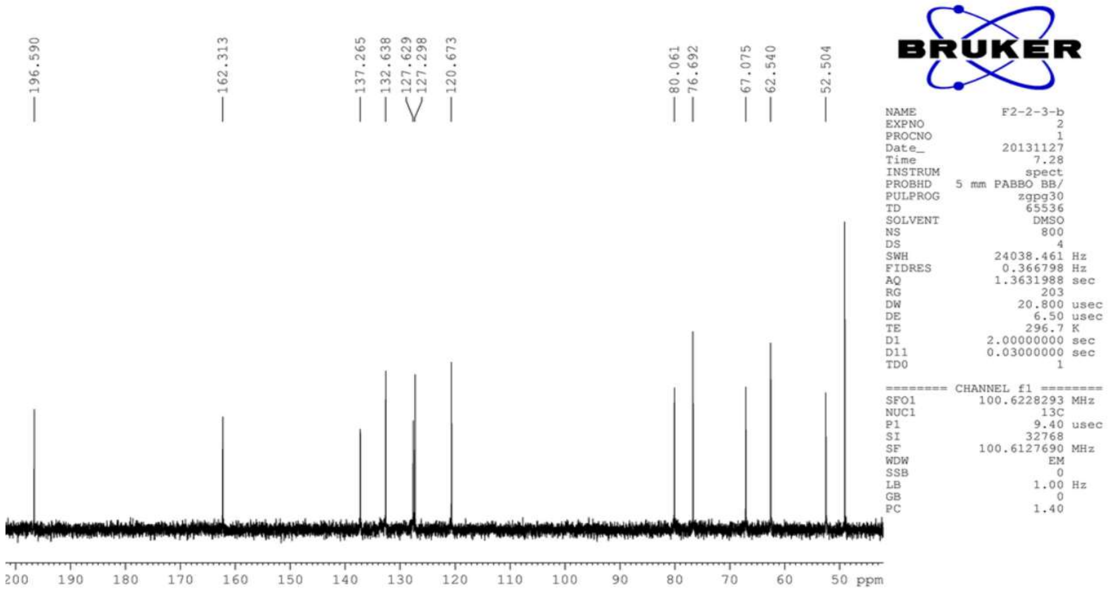

Figure S5. The ^{1}H-NMR (600 MH*z*) and ^{13}C-NMR (150 MH*z*) spectrums of compound **2** in DMSO-d_6.

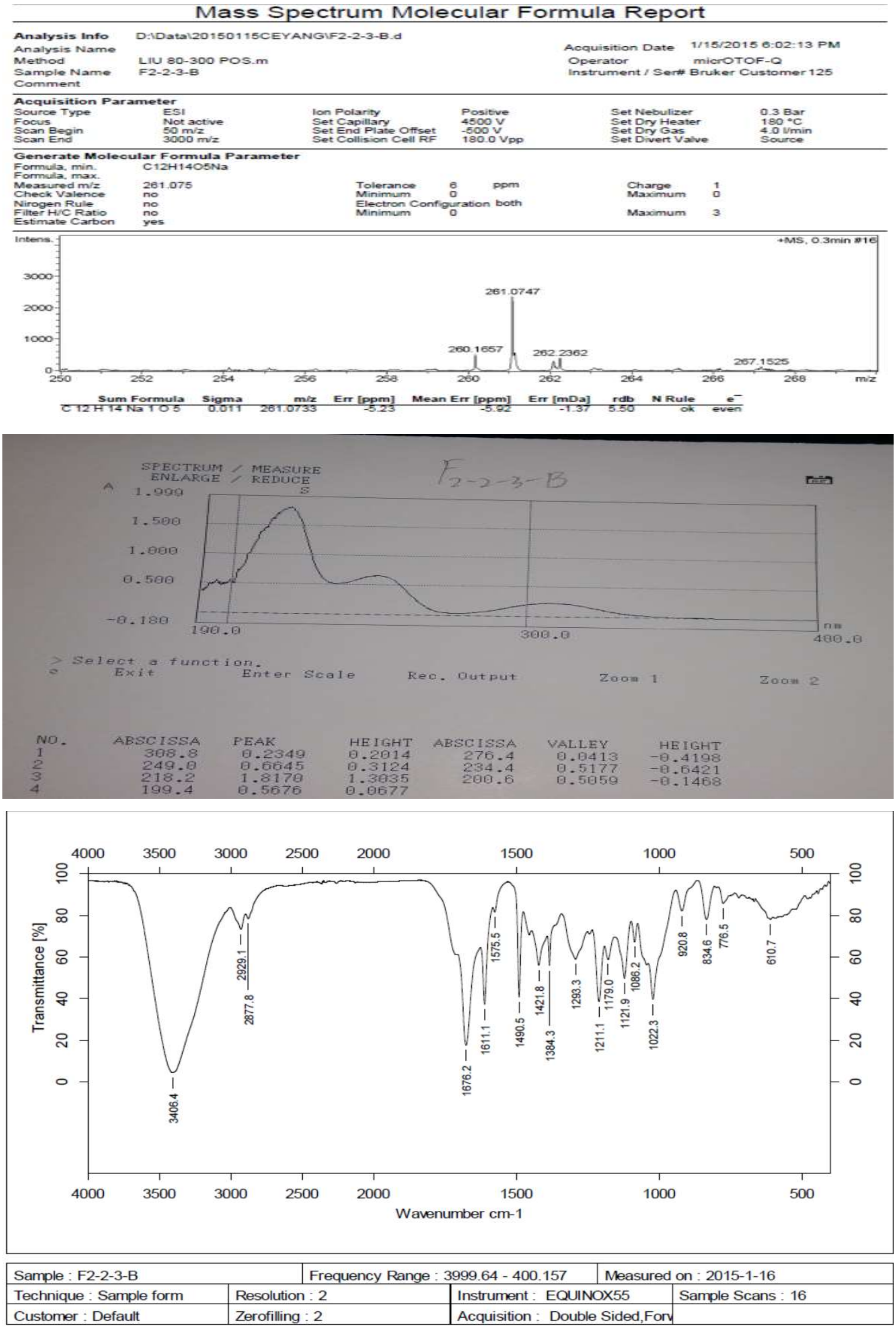

Figure S6. The HR-ESI-MS, UV and IR spectrums of compound **2**.

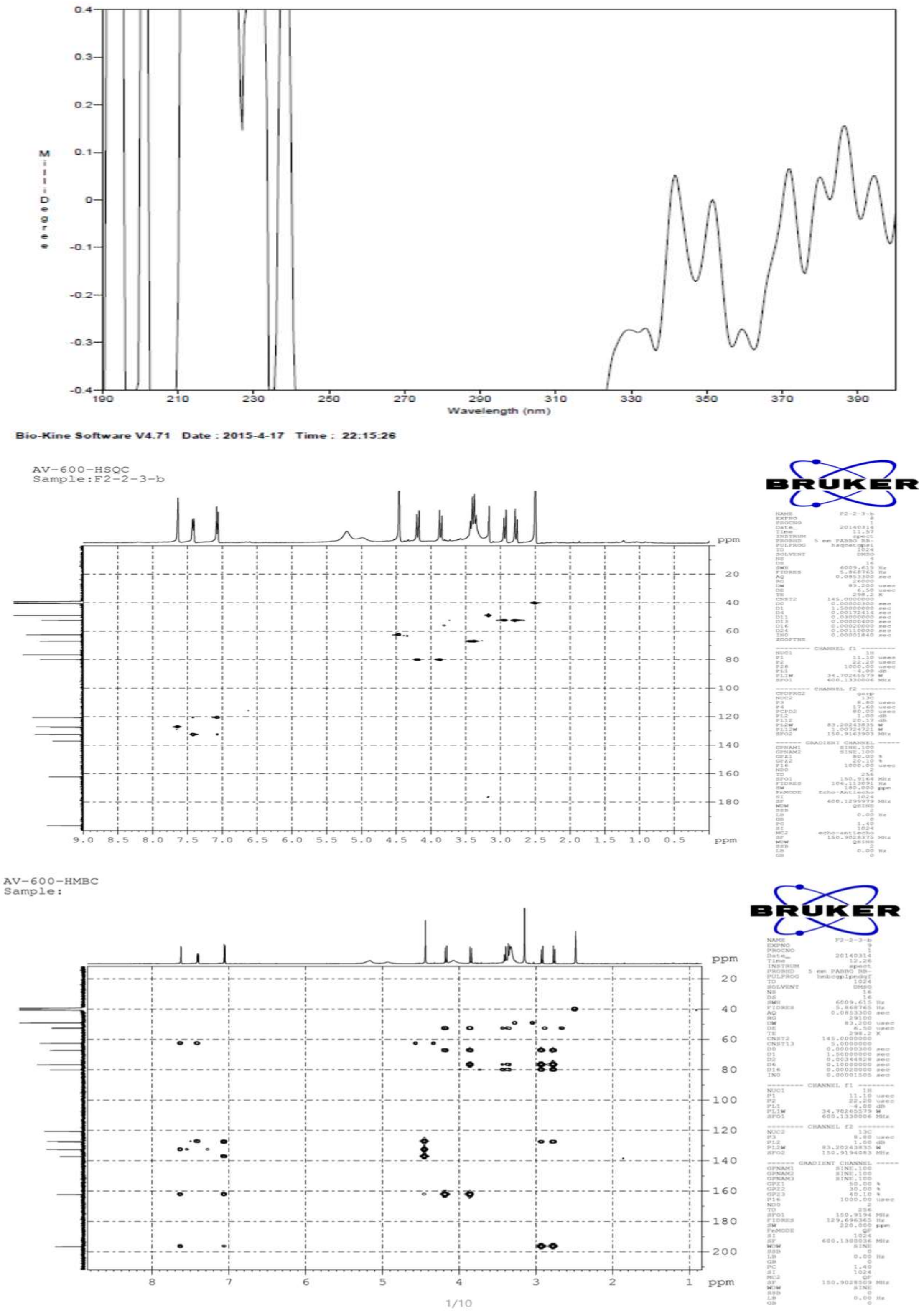

Figure S7. The CD, HSQC (600 MH*z*) and HMBC (600 MH*z*) spectrums of compound **2**.

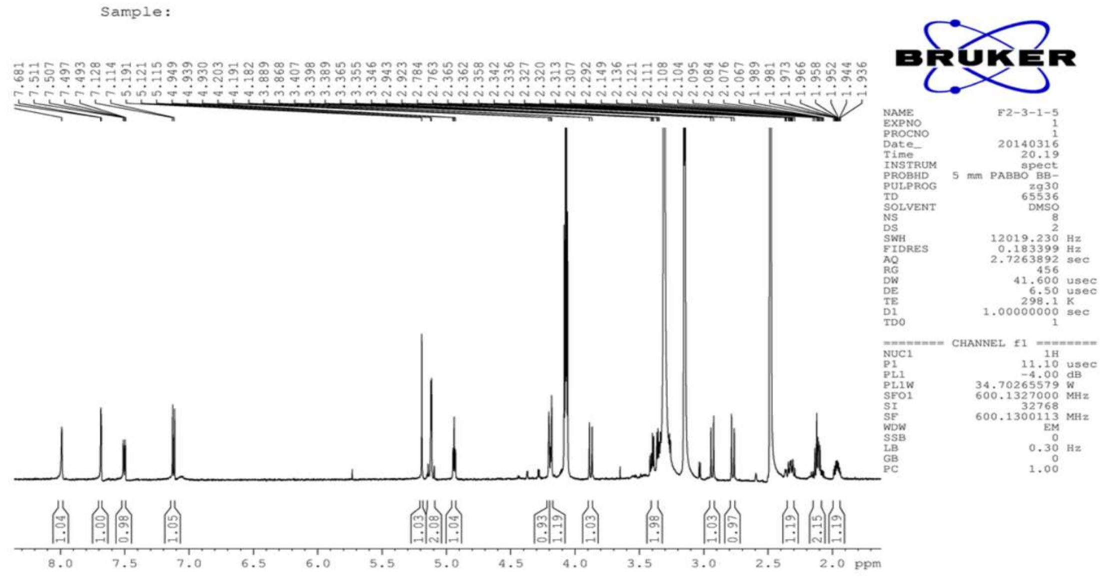

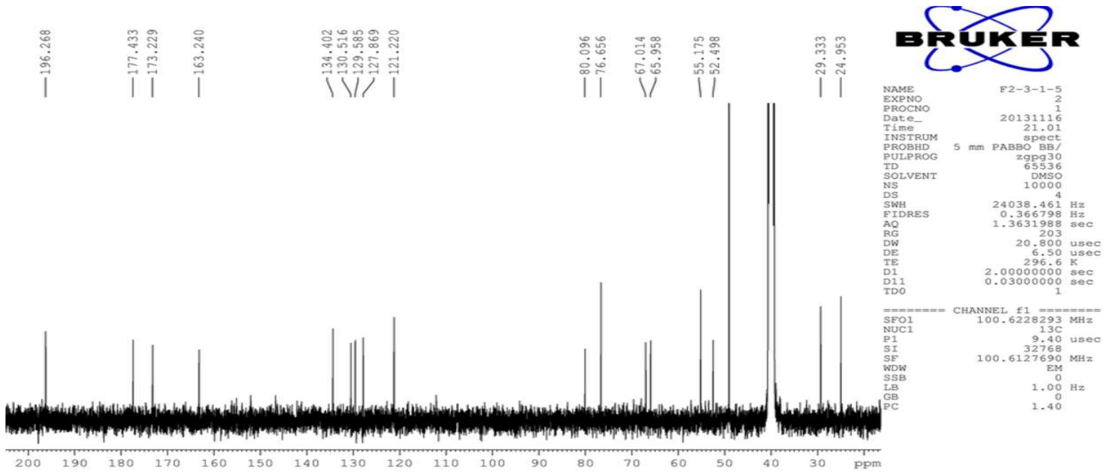

Figure S8. The ^{1}H-NMR (600 MH*z*) and ^{13}C-NMR (150 MH*z*) spectrums of compound **3** in DMSO-d_6.

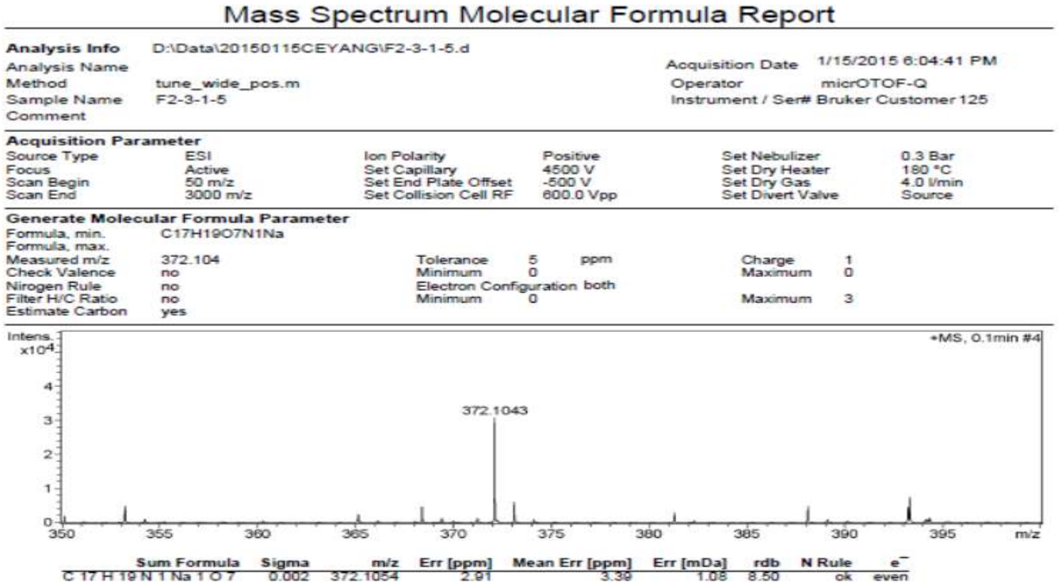

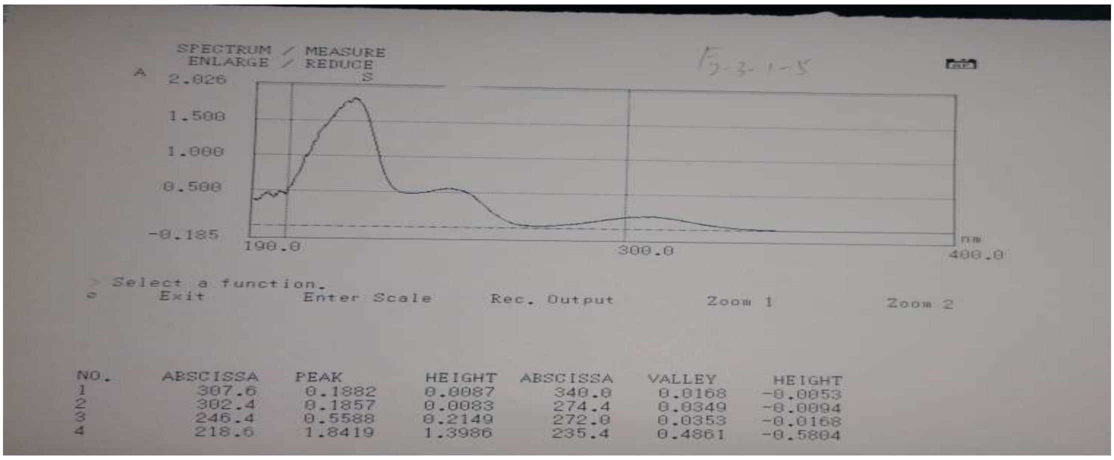

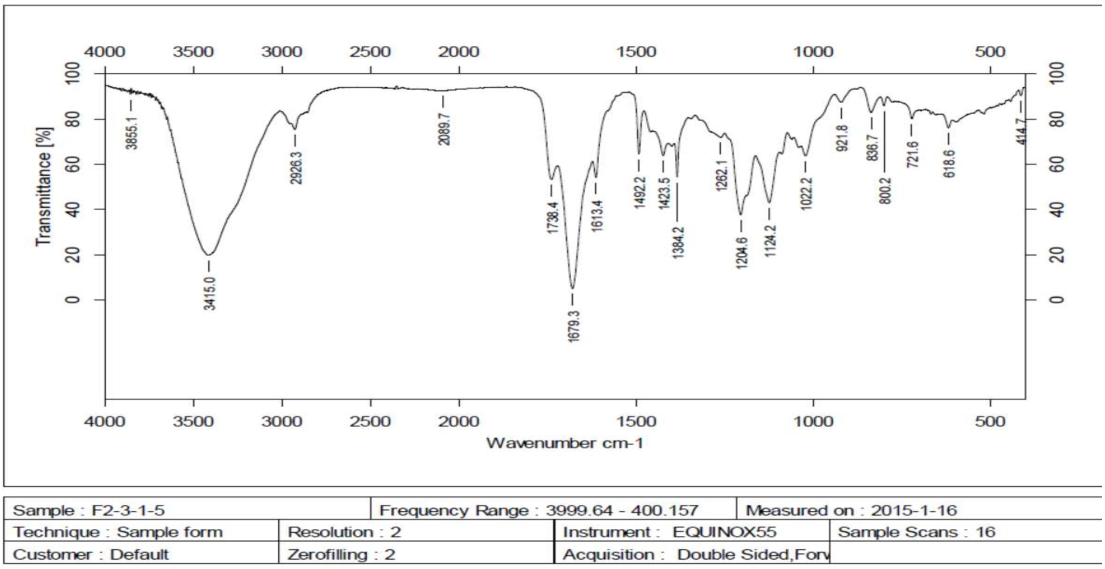

Figure S9. The HR-ESI-MS, UV and IR spectrums of compound **3**.

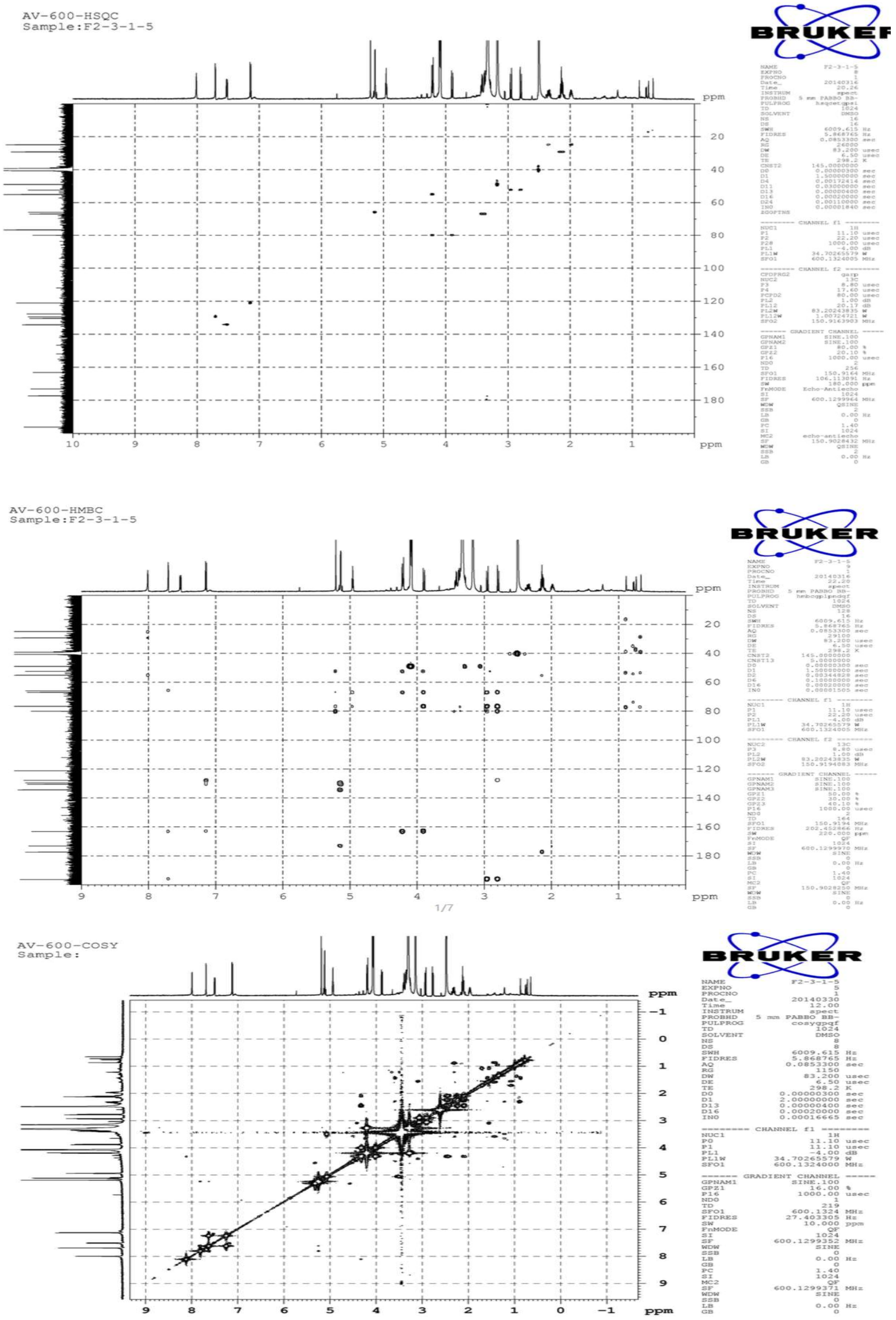

Figure S10. The HSQC, HMBC and ^{1}H-^{1}H COSY spectrums (600 MH*z*) of compound **3**.

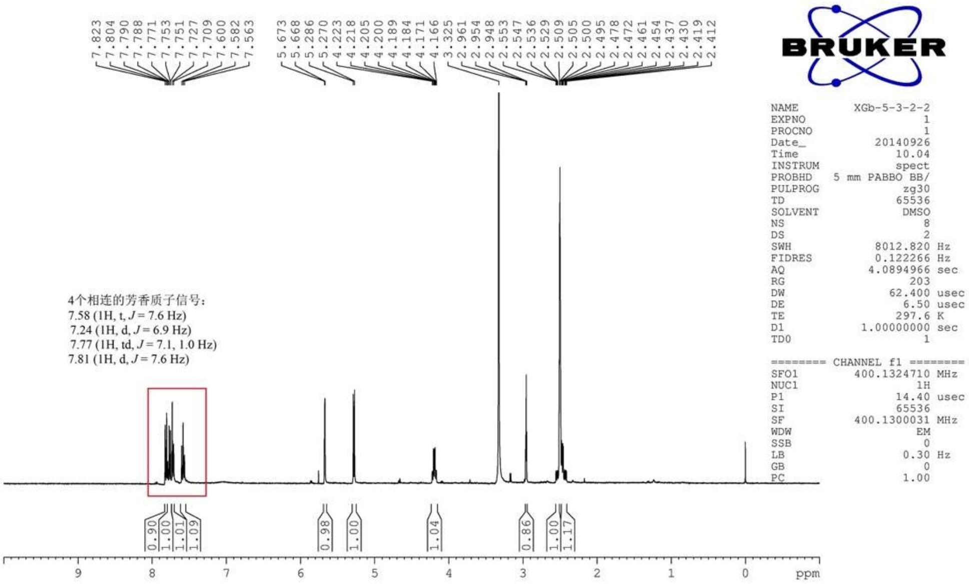

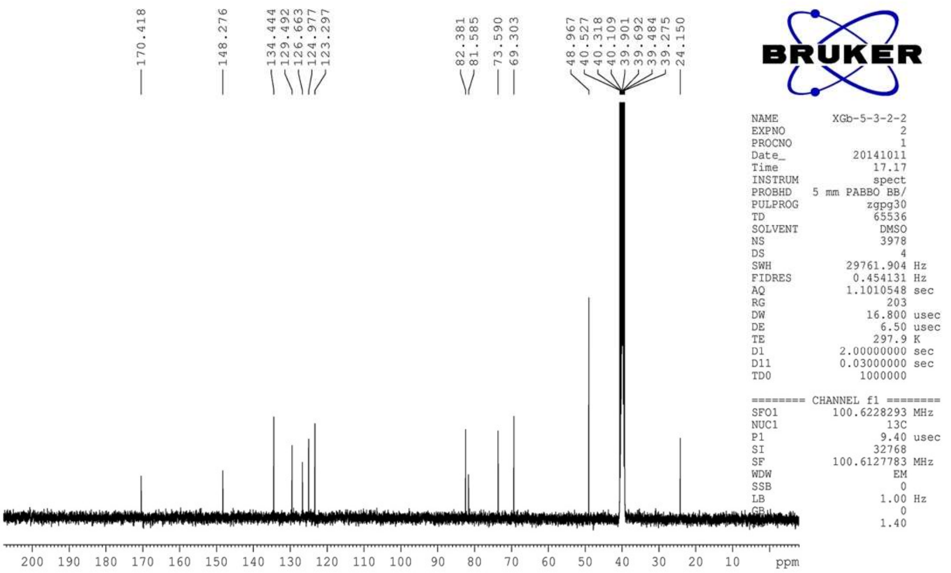

Figure S11. The ^{1}H-NMR (400 MH*z*) and ^{13}C-NMR (100 MH*z*) spectrums of compound **4** in DMSO-d_6 .

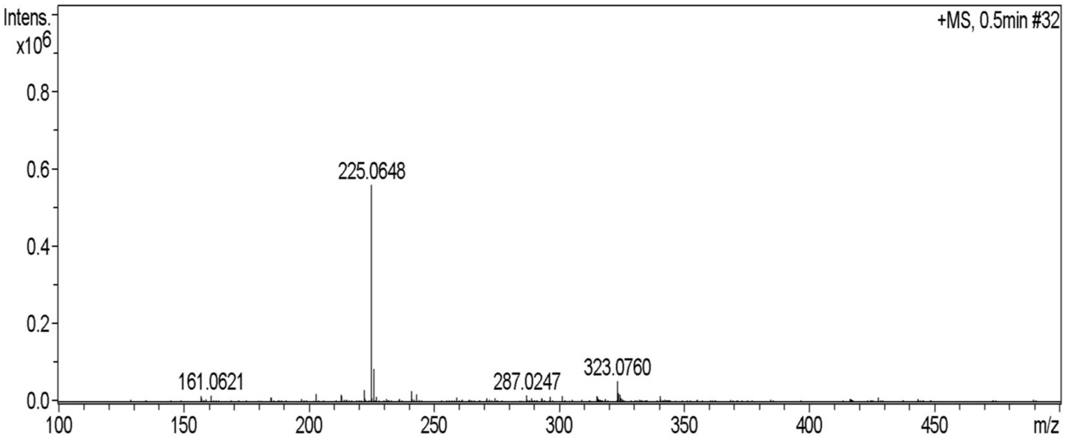

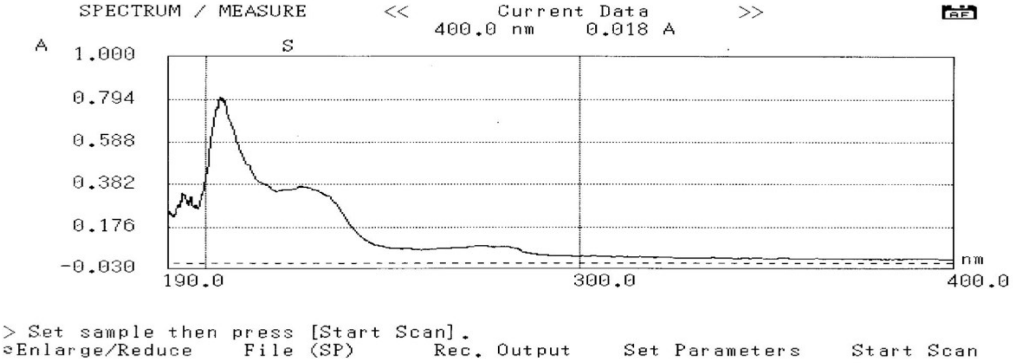

NO.	ABSCISSA	PEAK	HEIGHT	ABSCISSA	VALLEY	HEIGHT
1	279.6	0.0823	0.0122	257.4	0.0651	-0.1094
2	225.0	0.3730	0.0356	222.8	0.3559	-0.0559
3	203.6	0.8059	0.5123	198.0	0.2655	-0.2669
4	193.8	0.3412	0.0847			

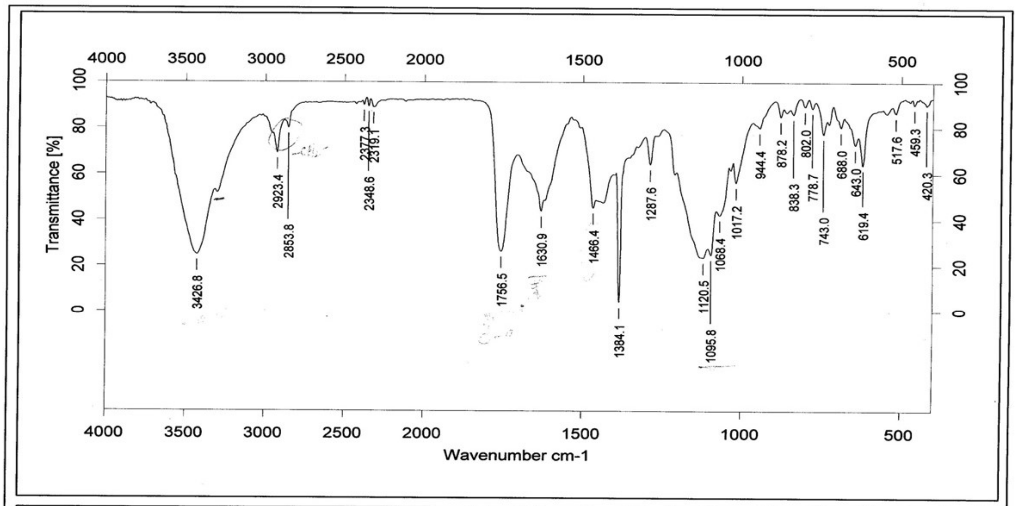

Sample : b-5-3-2-2	Frequency Range : 3999.64 - 400.157		Measured on : 2015-4-24
Technique : Sample form	Resolution : 2	Instrument : EQUINOX55	Sample Scans : 16
Customer : Default	Zerofilling : 2	Acquisition : Double Sided,For	

Figure S12. The HR-ESI-MS, UV and IR spectrums of compound **4**.

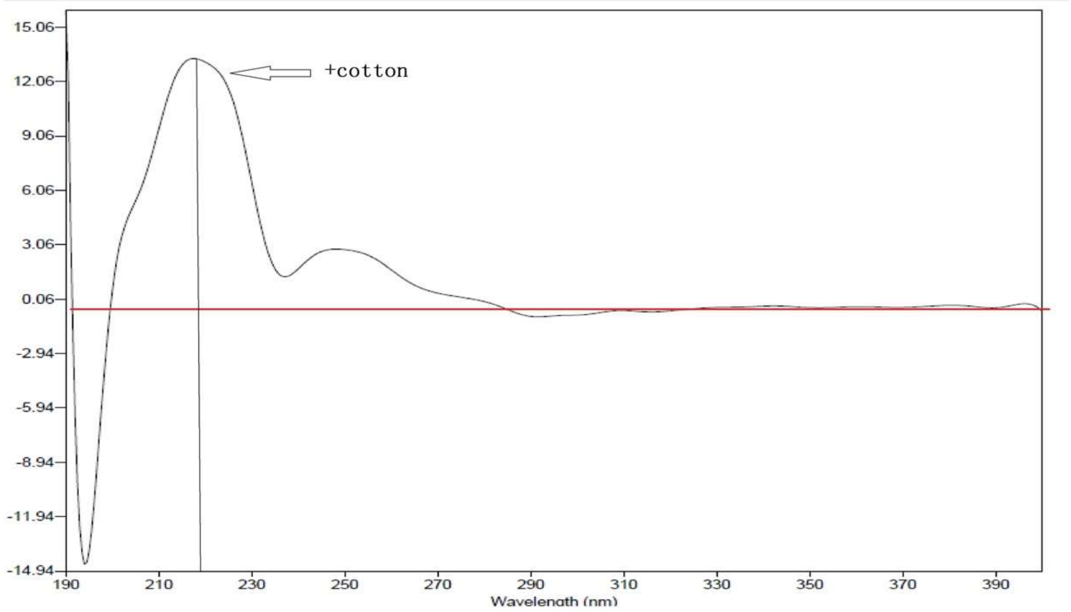

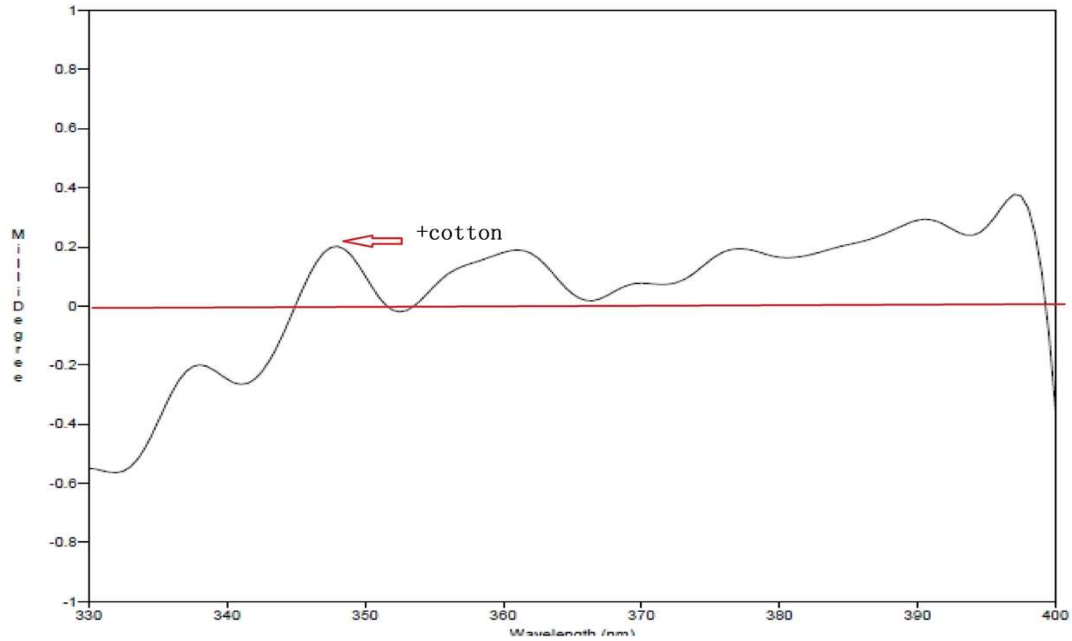

Figure S13. The CD spectrum of compound **4**.

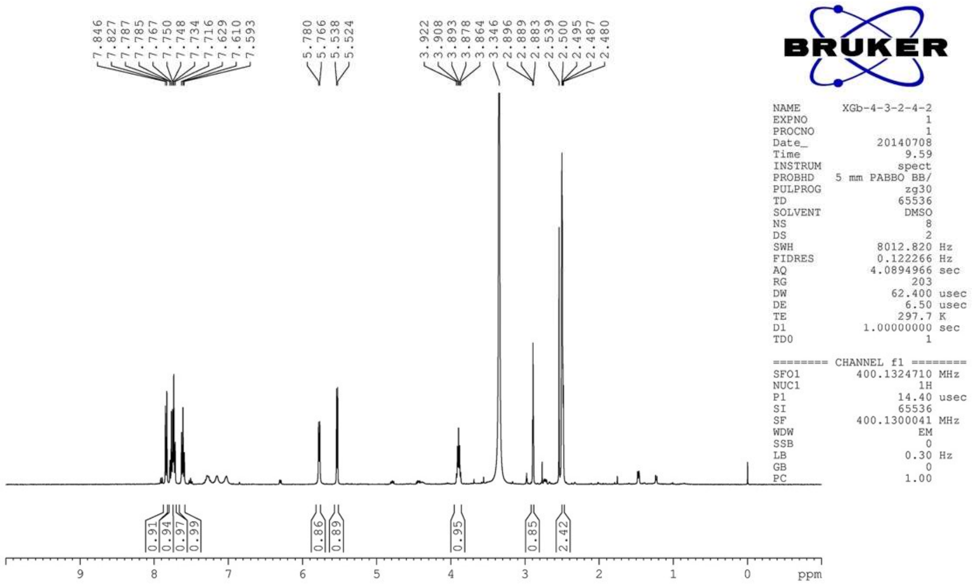

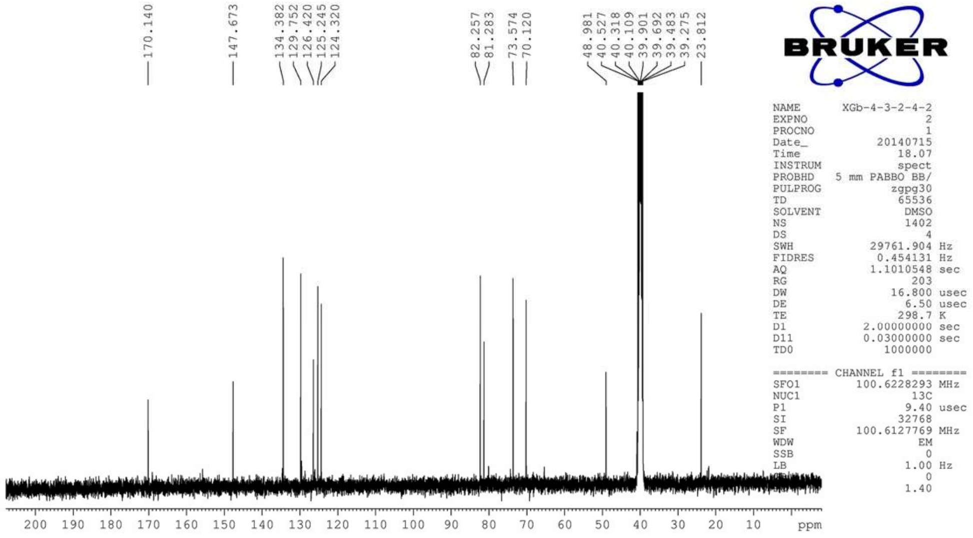

Figure S14. The ^{1}H-NMR (400 MH*z*) and ^{13}C-NMR (100 MH*z*) spectrums of compound **5** in DMSO-d_6 .

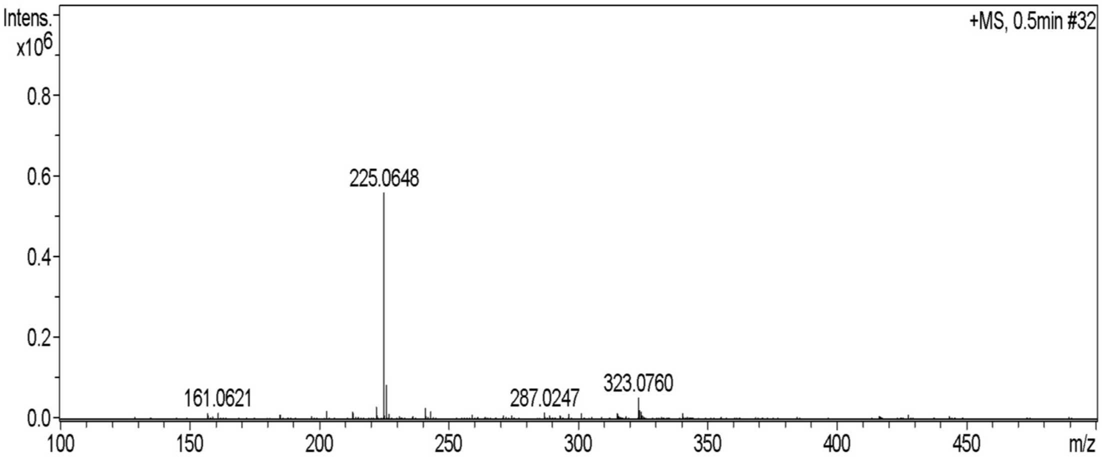

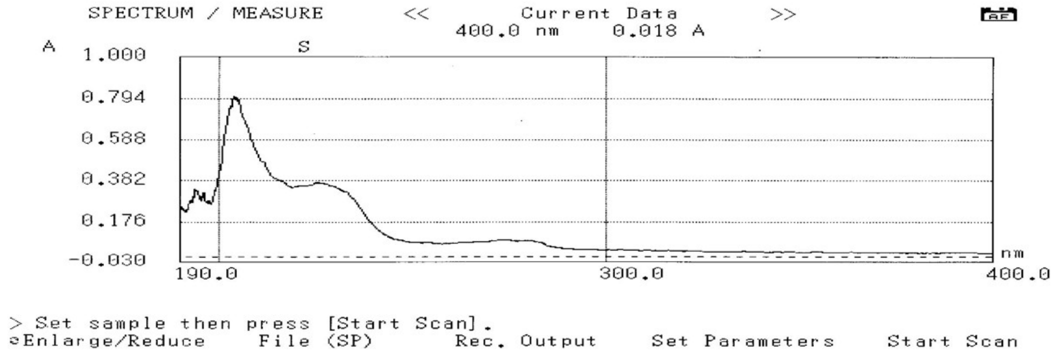

NO.	ABSCISSA	PEAK	HEIGHT	ABSCISSA	VALLEY	HEIGHT
1	279.6	0.0823	0.0122	257.4	0.0651	-0.1094
2	225.0	0.3730	0.0356	222.8	0.3559	-0.0559
3	203.6	0.8059	0.5123	198.0	0.2655	-0.2669
4	193.8	0.3412	0.0847			

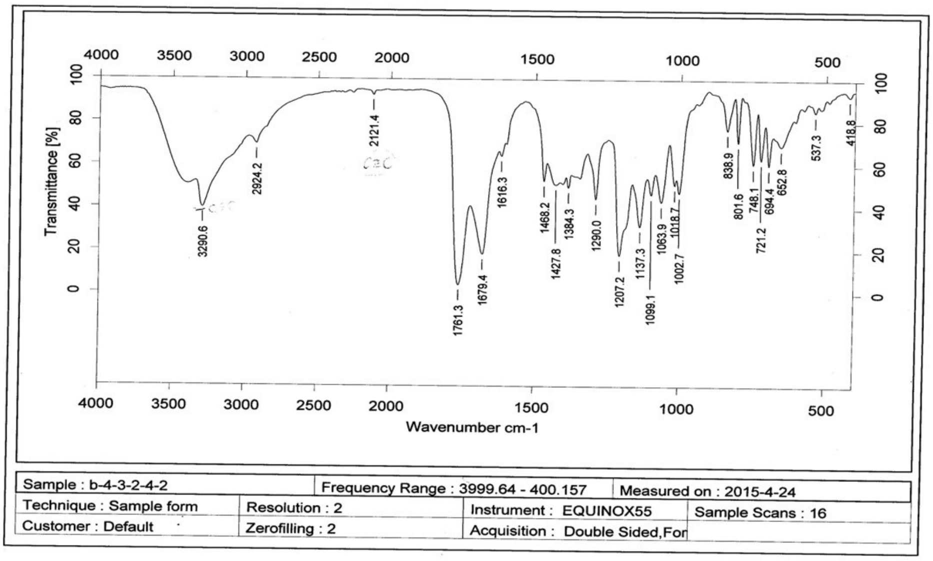

Figure S15. The HR-ESI-MS, UV and IR spectrums of compound **5**.

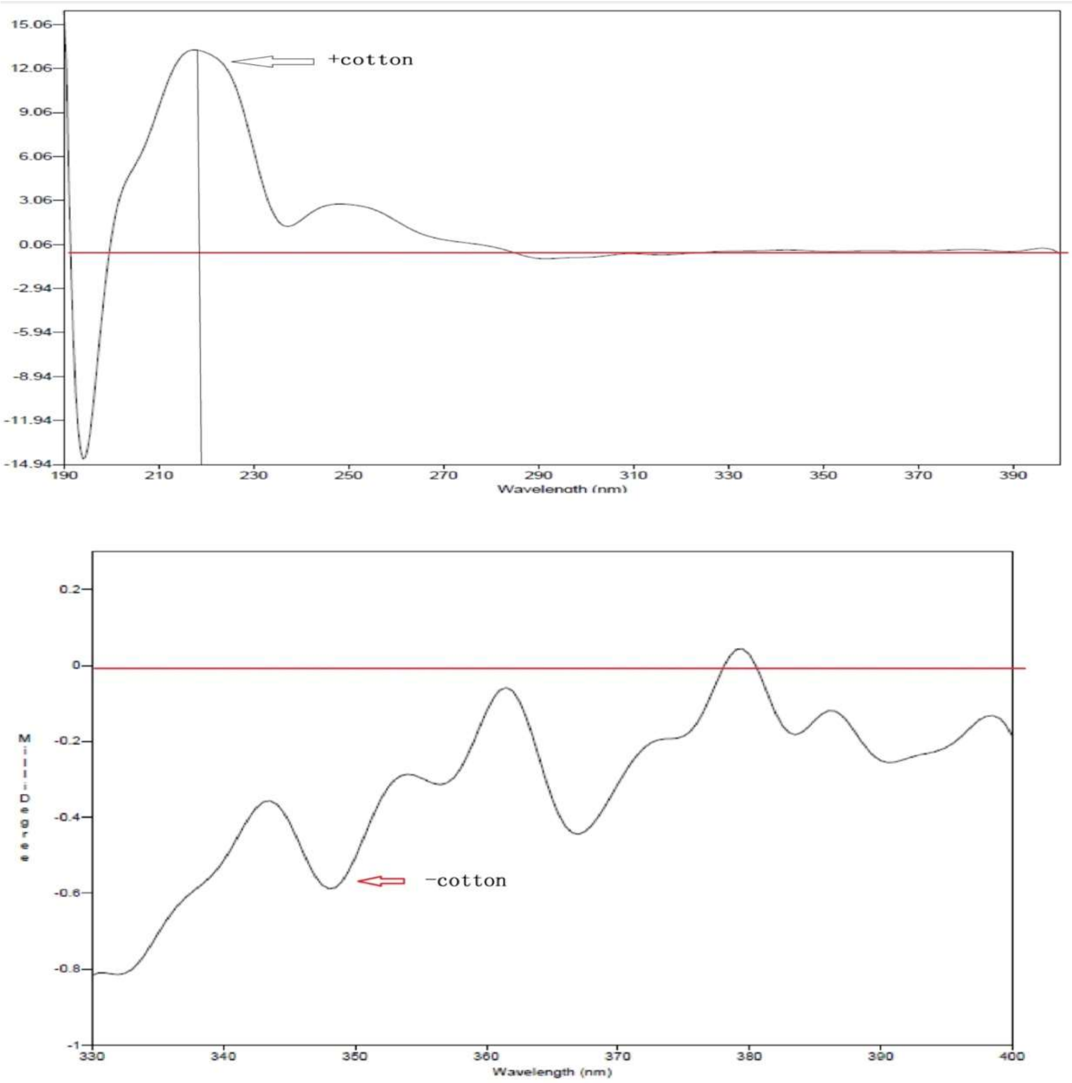

Figure S16. The CD spectrum of compound **5**.

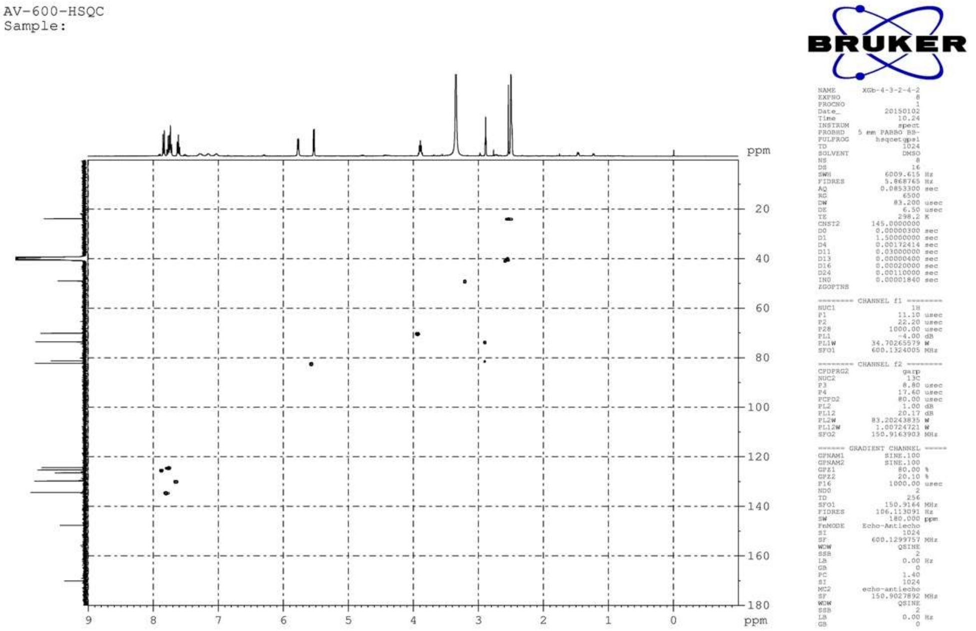

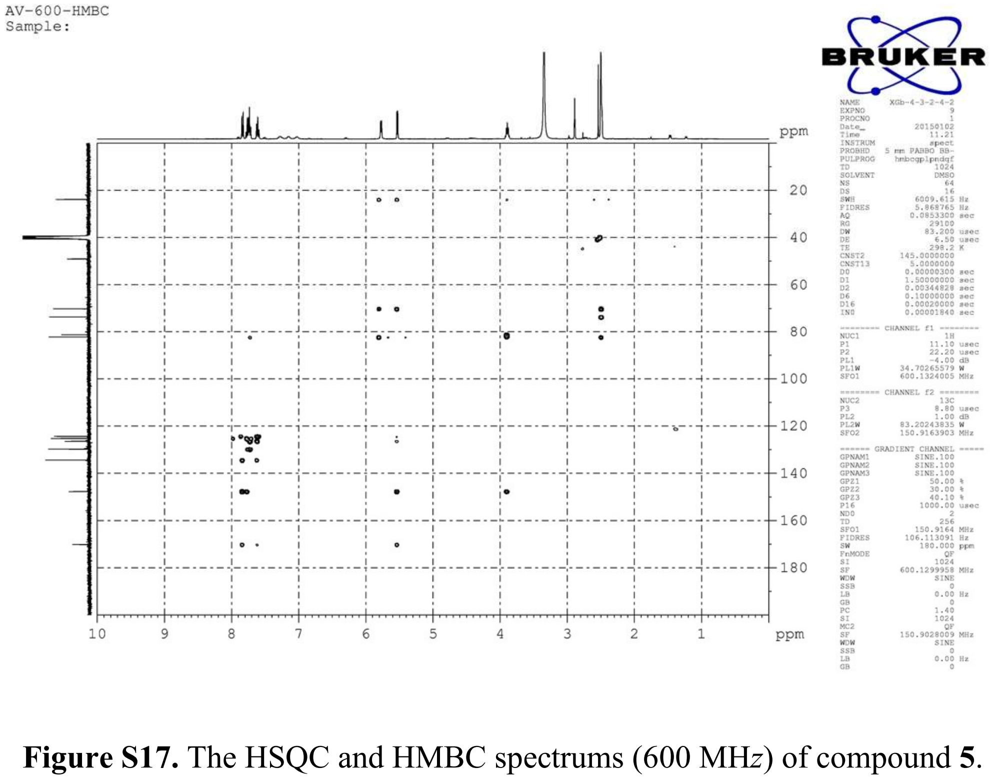

Figure S17. The HSQC and HMBC spectrums (600 MH*z*) of compound **5**.

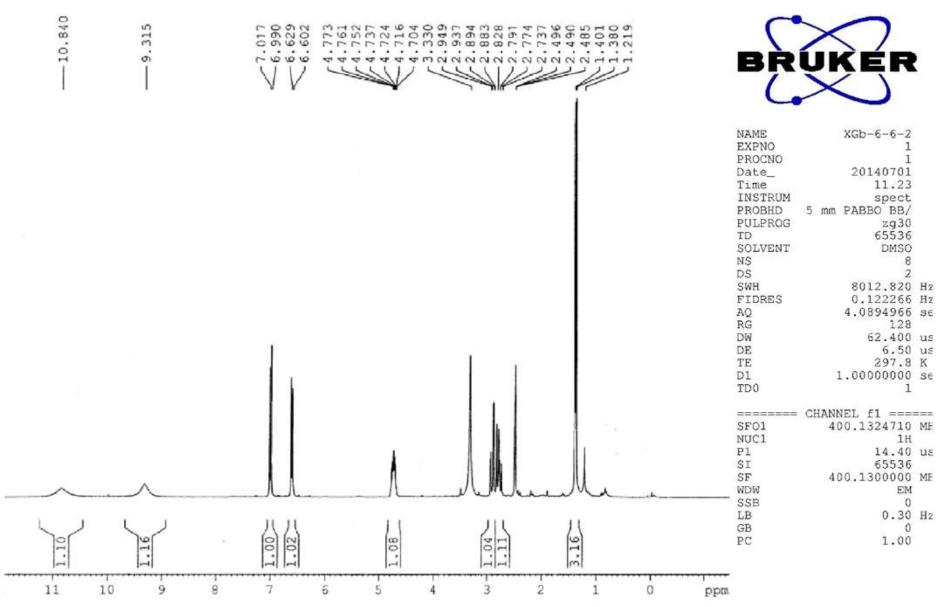

Figure S18. The ^{1}H-NMR (400 MH*z*) spectrum of compound **6** in DMSO-d_6.

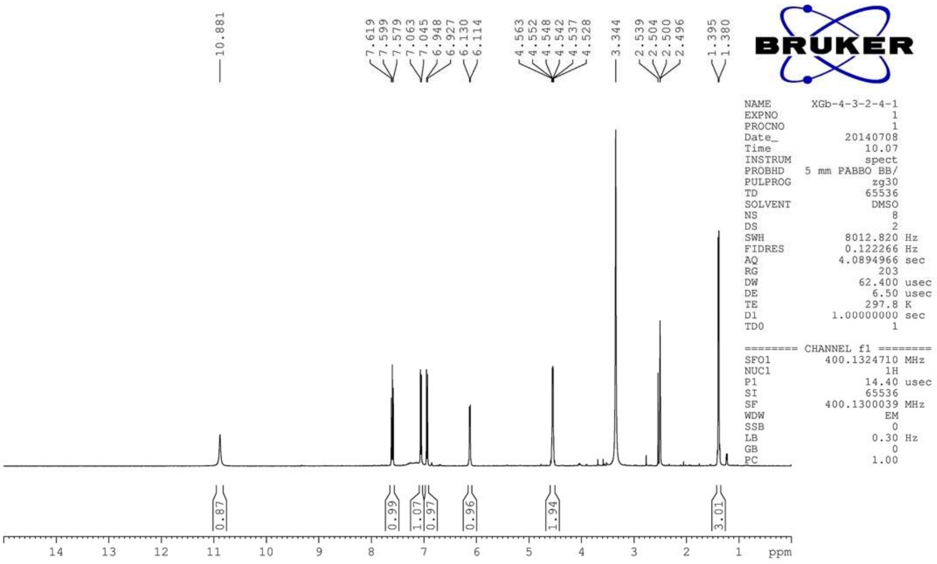

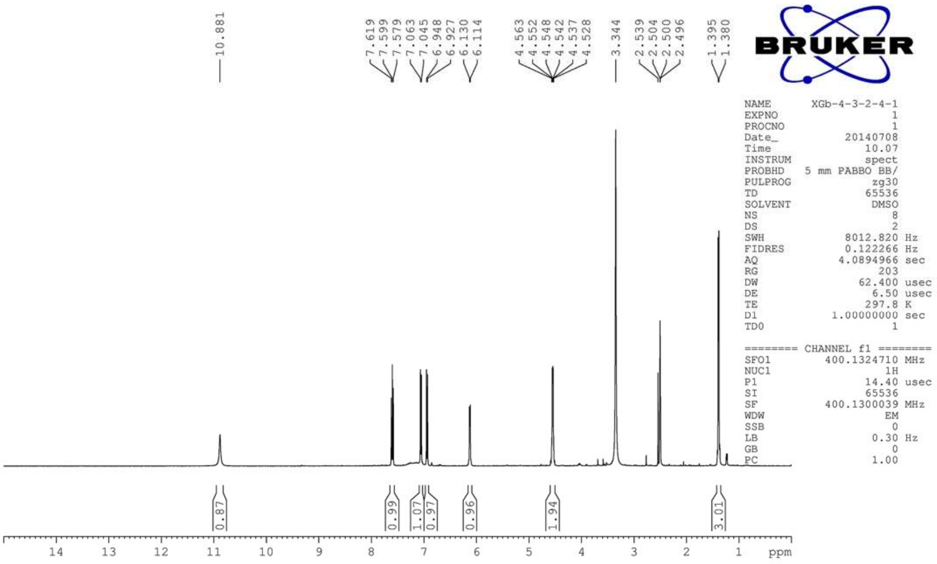

Figure S19. The ^{1}H-NMR (400 MHz) and ^{13}C-NMR (100 MHz) spectrums of compound **7** in DMSO-d_6 .

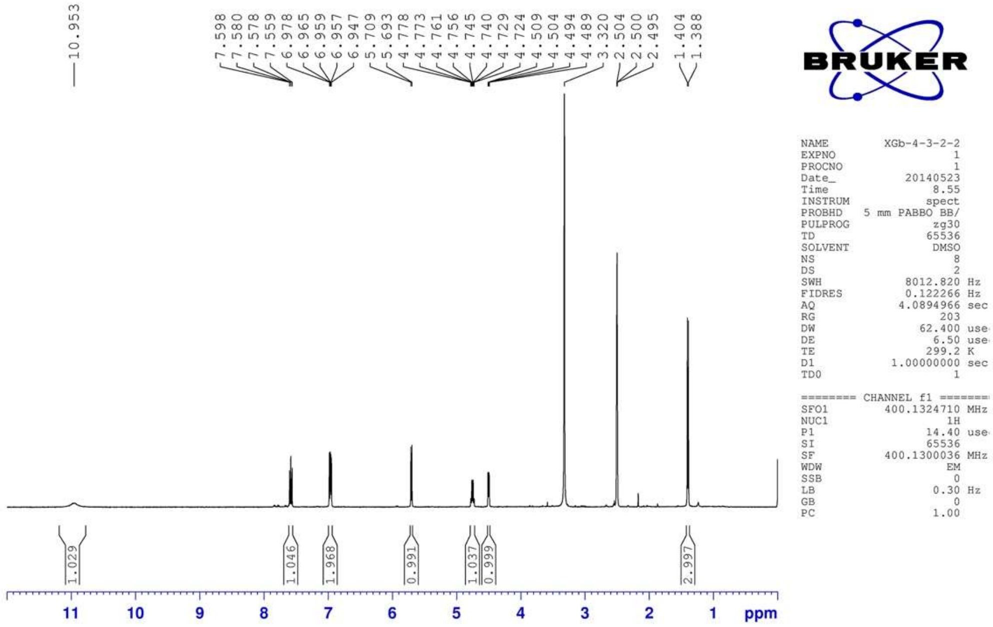

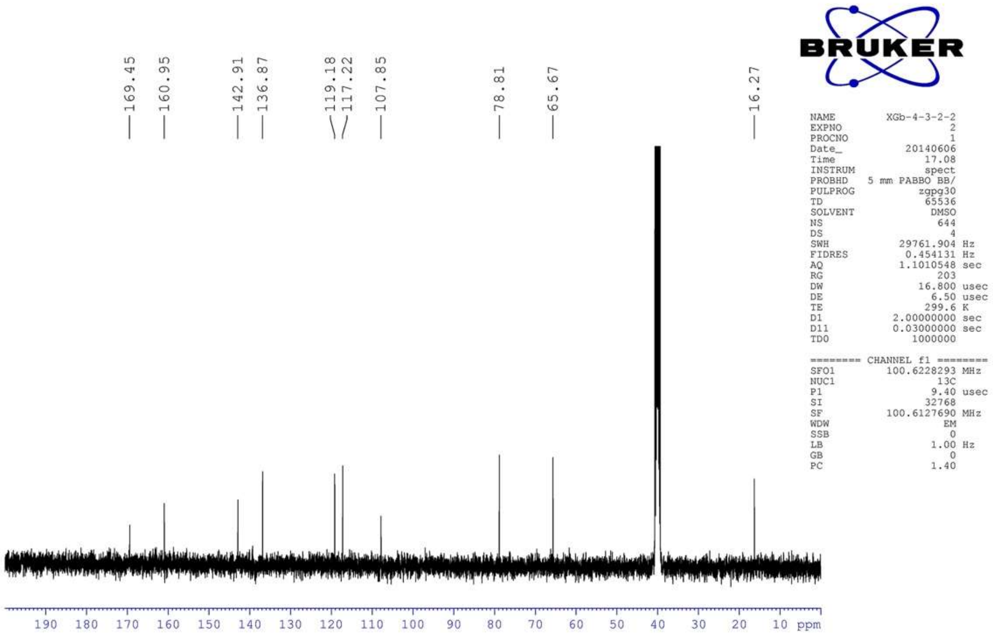

Figure S20. The ^{1}H-NMR (400 MH*z*) and ^{13}C-NMR (100 MH*z*) spectrums of compound **8** in DMSO-d_6.

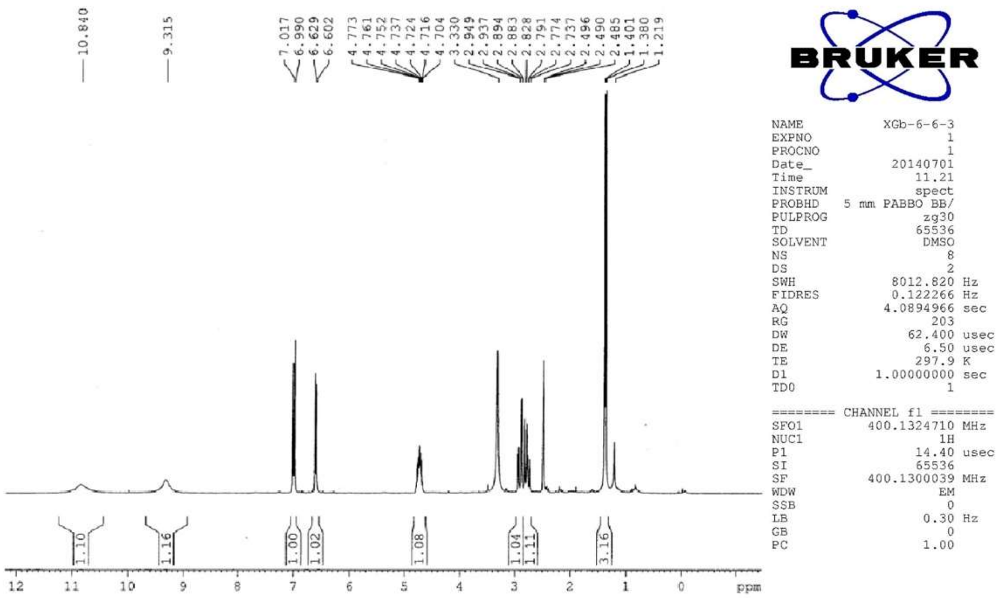

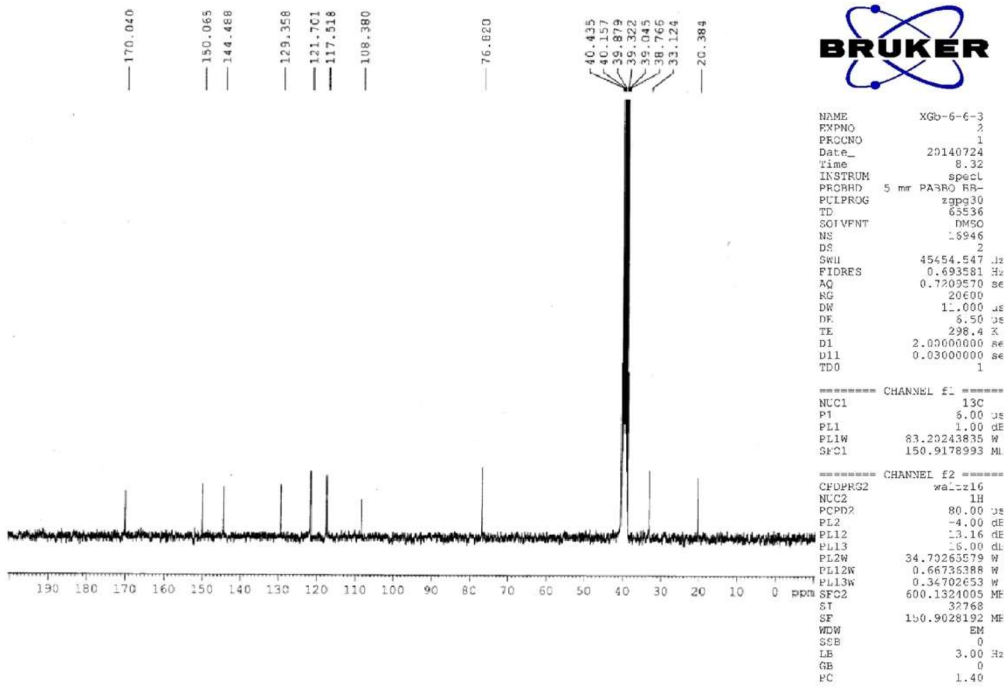

Figure S21. The ^{1}H-NMR (400 MH*z*) and ^{13}C-NMR (100 MH*z*) spectrums of compound **9** in DMSO-d_6.

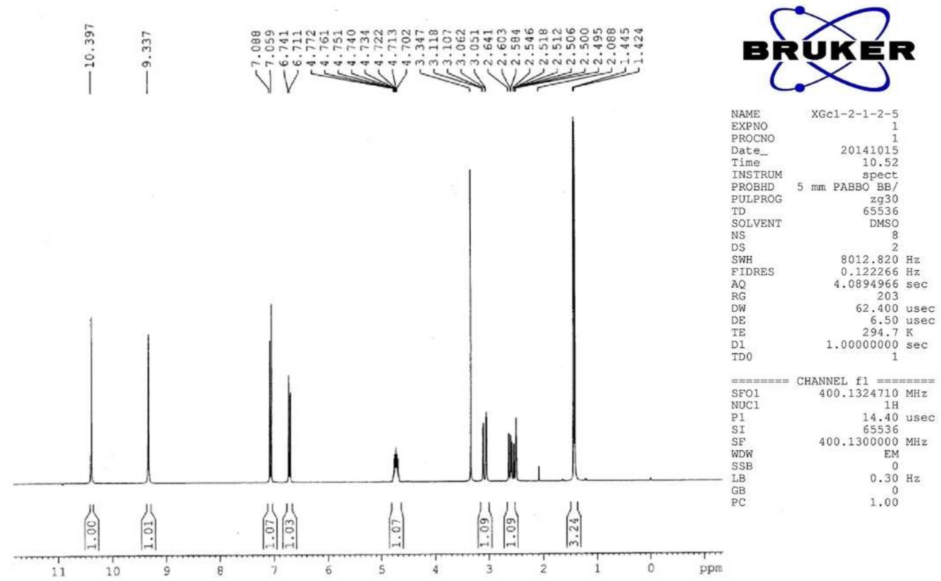

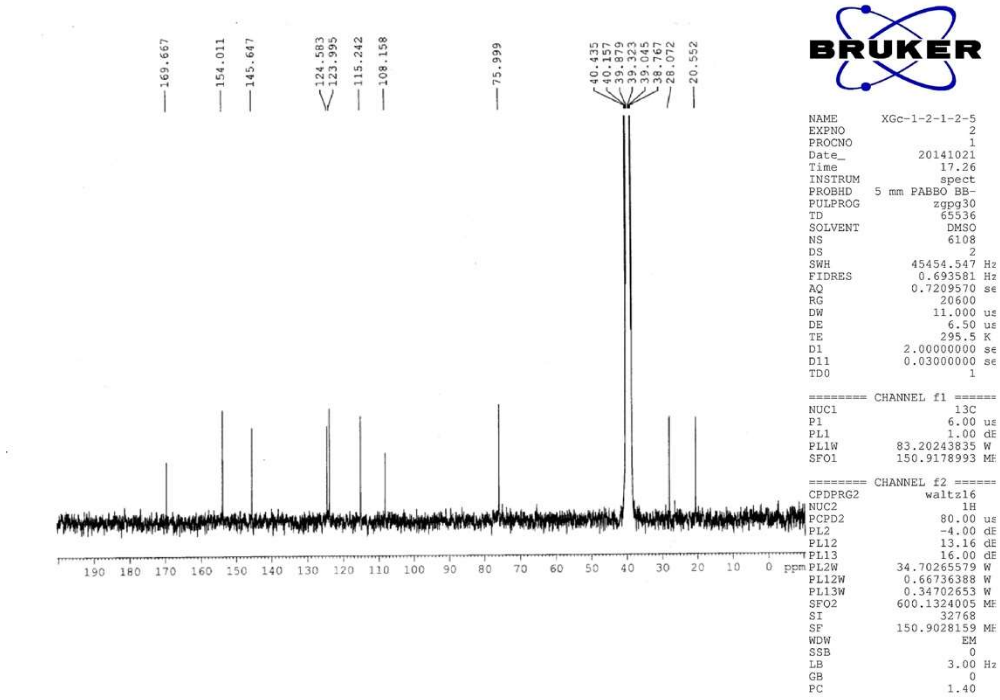

Figure S22. The ^{1}H-NMR (400 MH*z*) and ^{13}C-NMR (100 MH*z*) spectrums of compound **10** in DMSO-d_6.

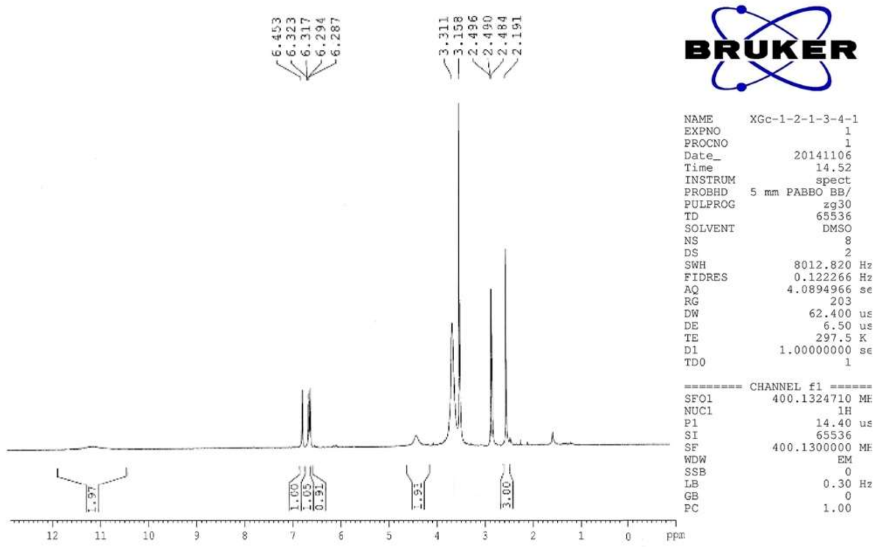

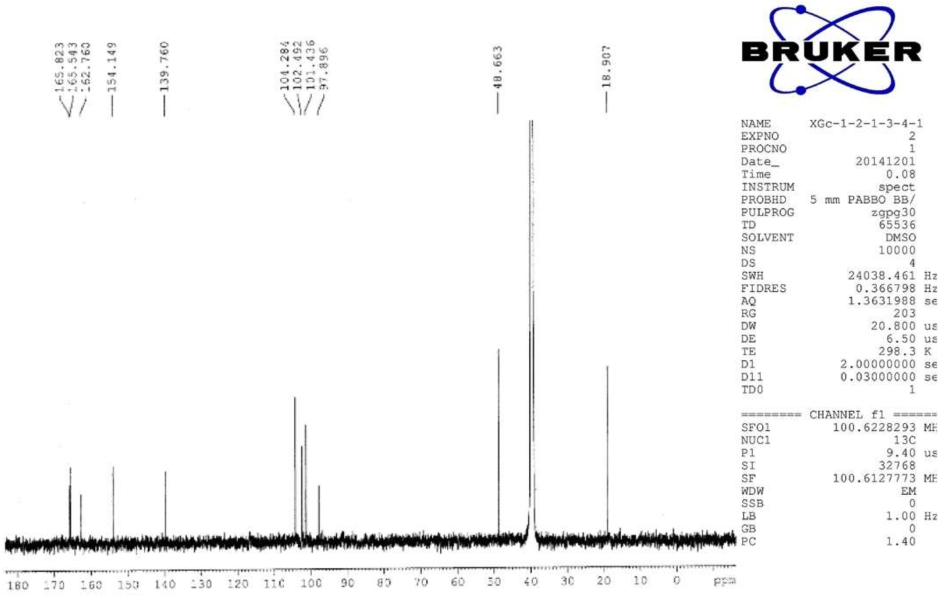

Figure S23. The ^{1}H-NMR (400 MH*z*) and ^{13}C-NMR (100 MH*z*) spectrums of compound **11** in DMSO-d_6.

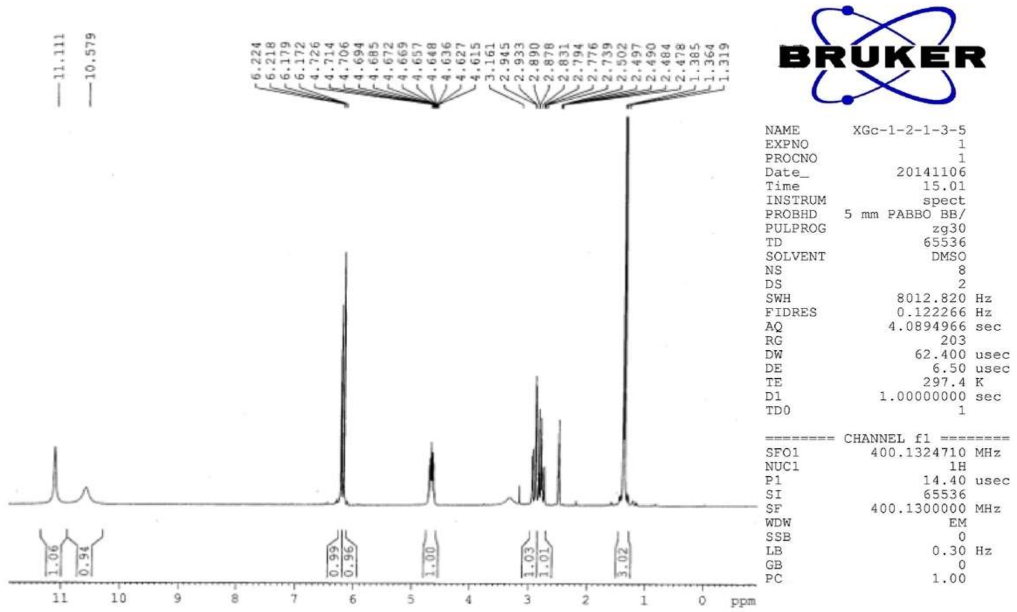

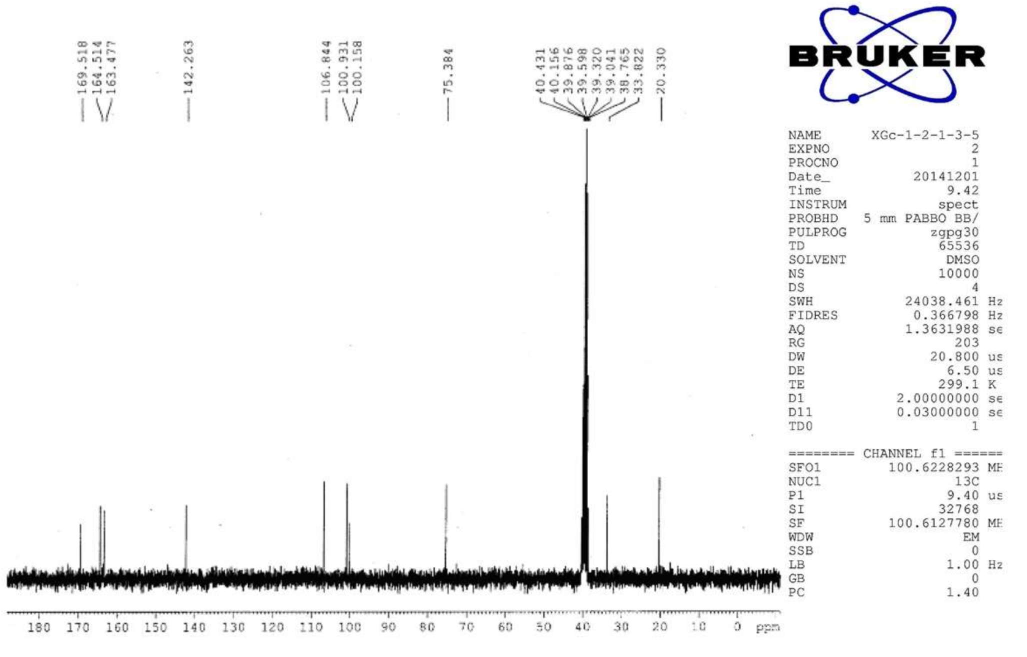

Figure S24. The ^{1}H-NMR (400 MH*z*) and ^{13}C-NMR (100 MH*z*) spectrums of compound **12** in DMSO-d_6.

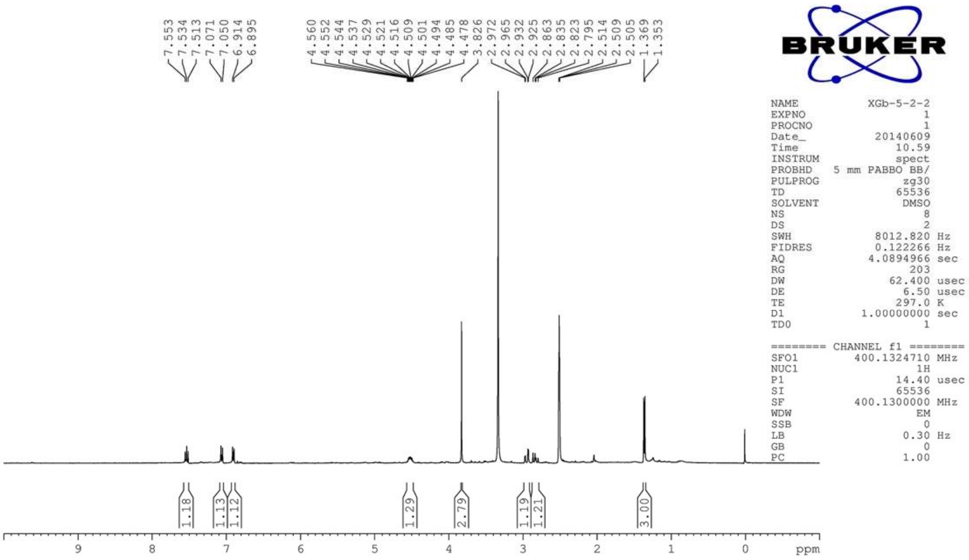

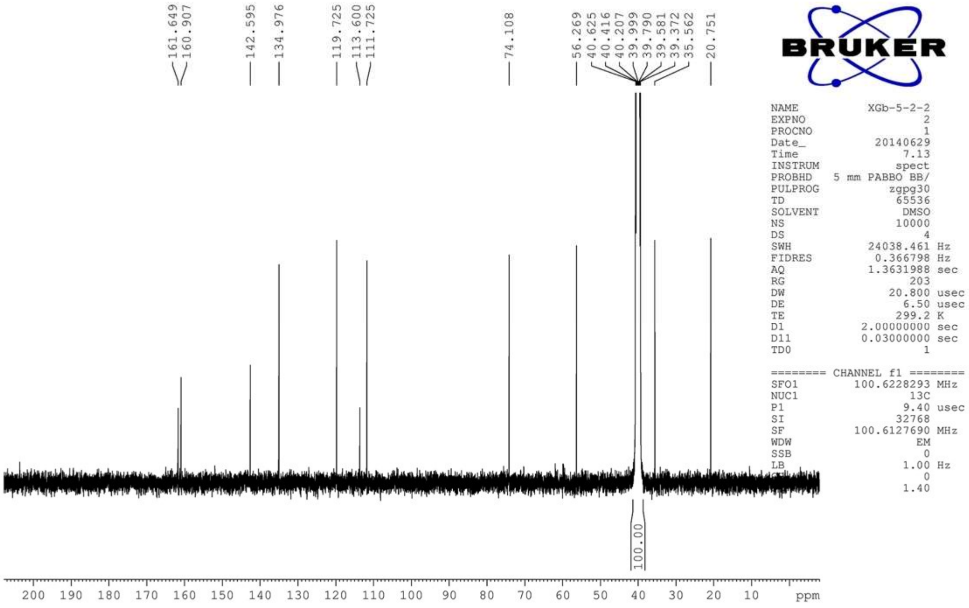

Figure S25. The ^{1}H-NMR (400 MH*z*) and ^{13}C-NMR (100 MH*z*) spectrums of compound **13** in DMSO-d_6.

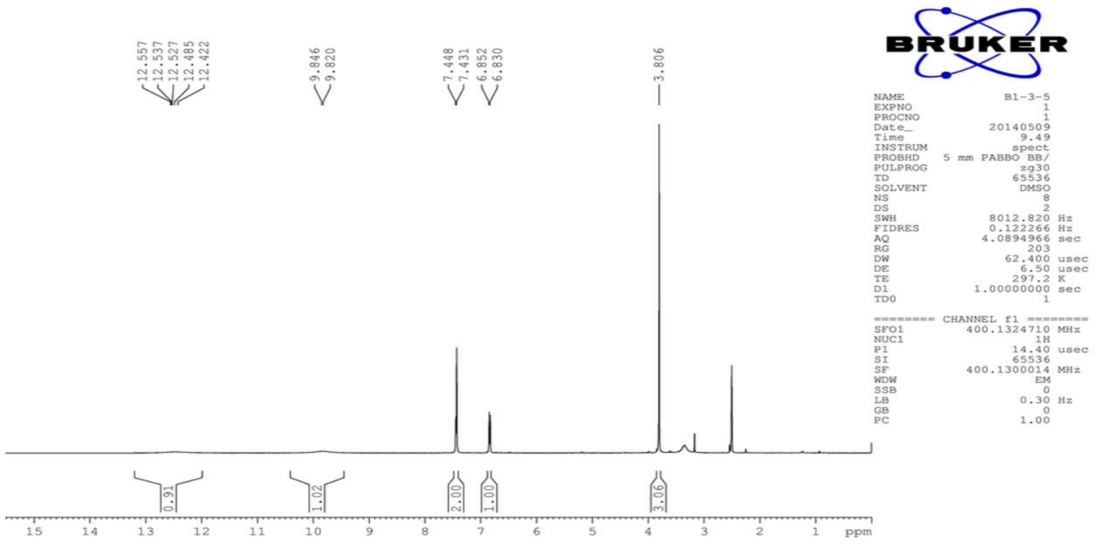

Figure S26. The ¹H-NMR (400 MH*z*) spectrum of compound **14** in DMSO-d_6.

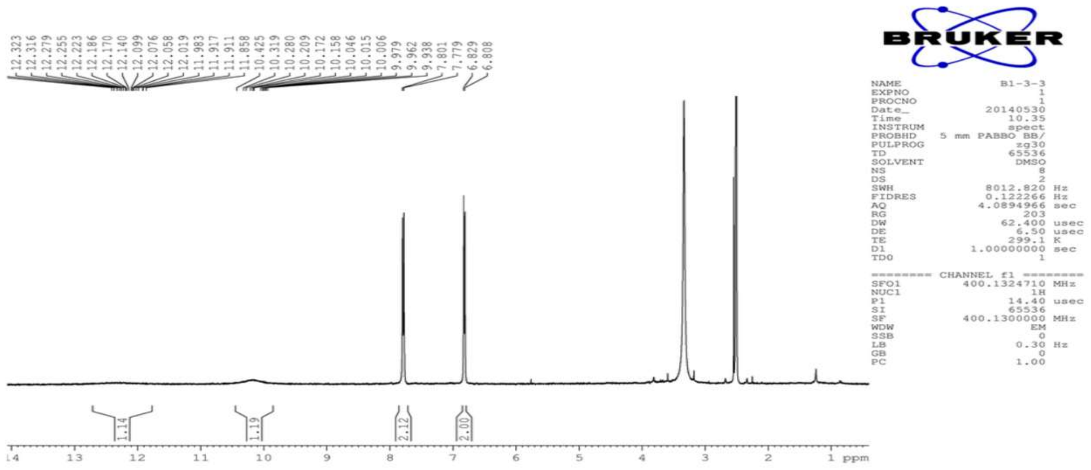

Figure S27. The ¹H-NMR (400 MH*z*) spectrum of compound **15** in DMSO-d_6.

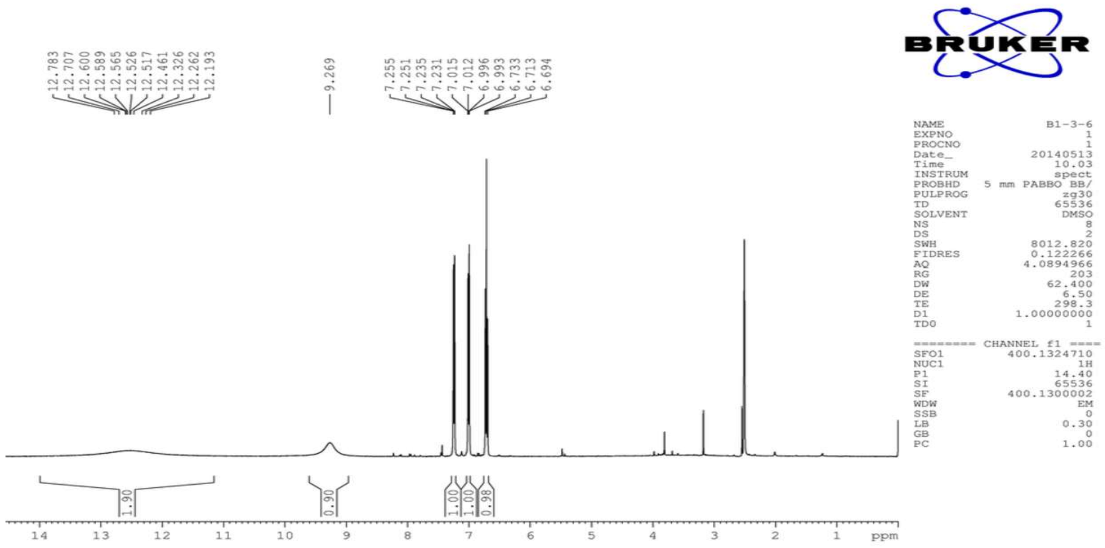

Figure S28. The ^{1}H-NMR (400 MH*z*) spectrum of compound **16** in DMSO-d_6.

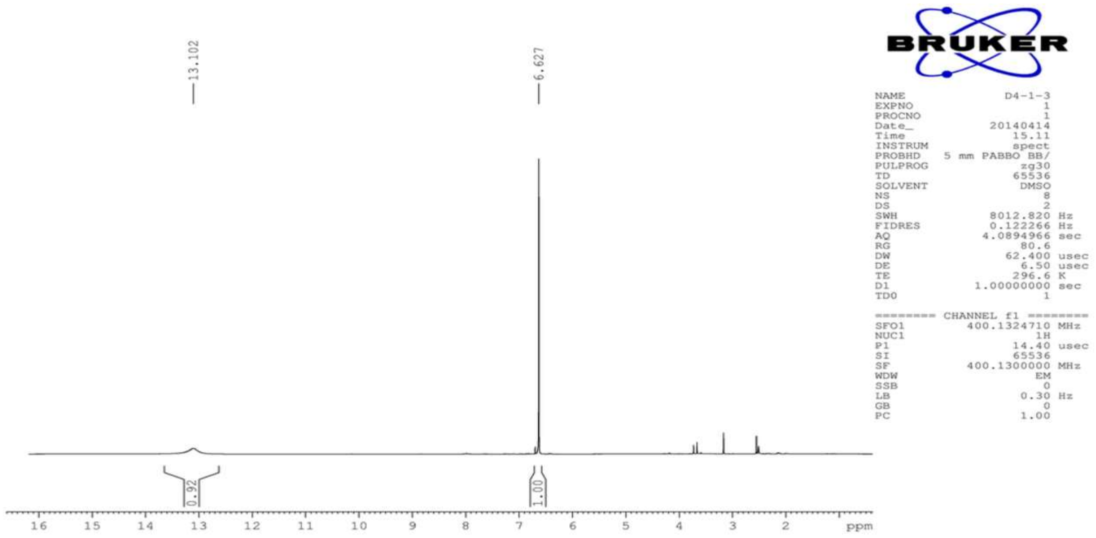

Figure S29. The ^{1}H-NMR (400 MH*z*) spectrum of compound **17** in DMSO-d_6.

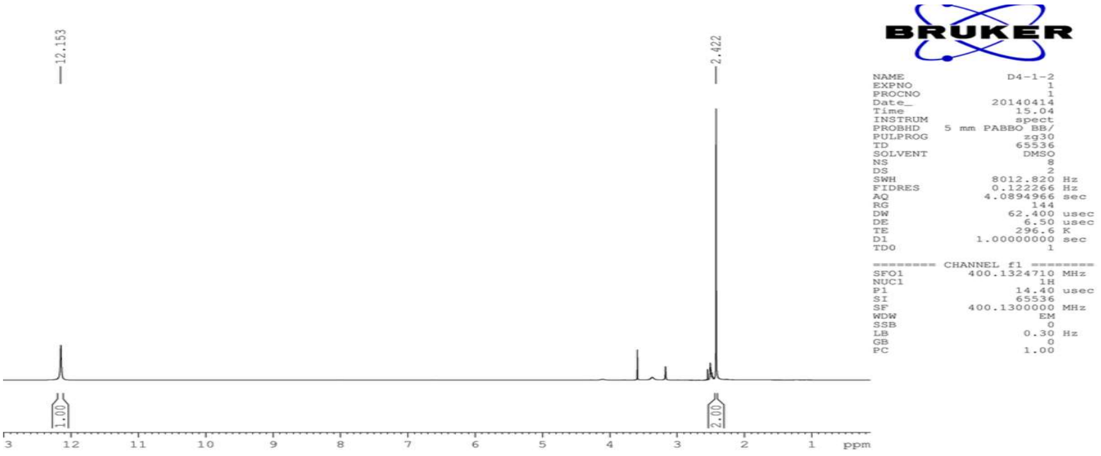

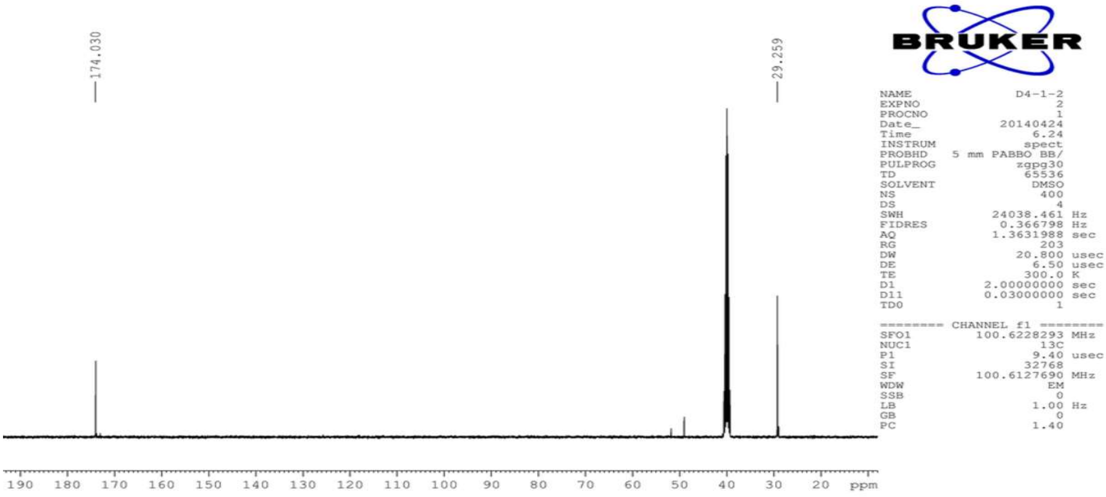

Figure S30. The ^{1}H-NMR (400 MH*z*) and ^{13}C-NMR (100 MH*z*) spectrums of compound **18** in DMSO-d_6.

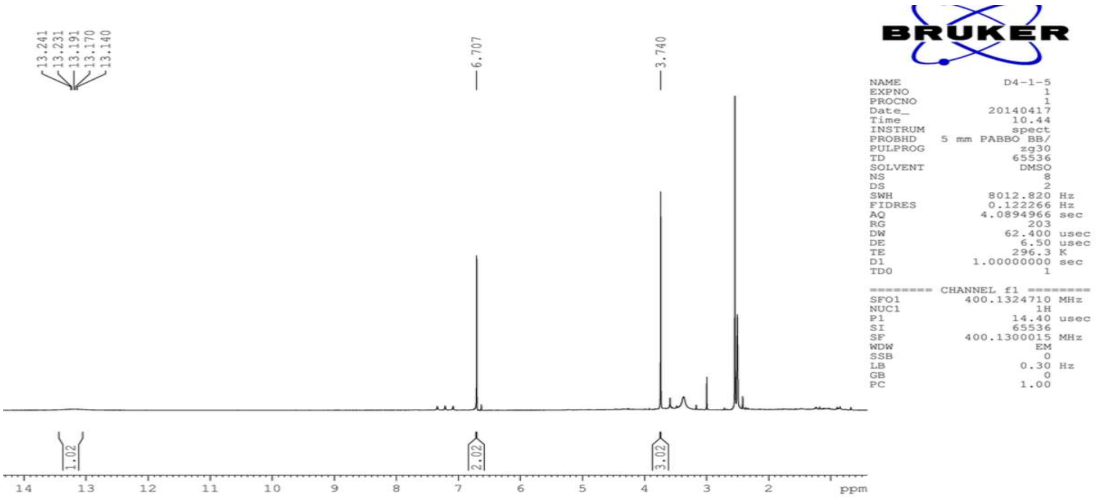

Figure S31. The ^{1}H-NMR (400 MH*z*) spectrum of compound **19** in DMSO-d_6.

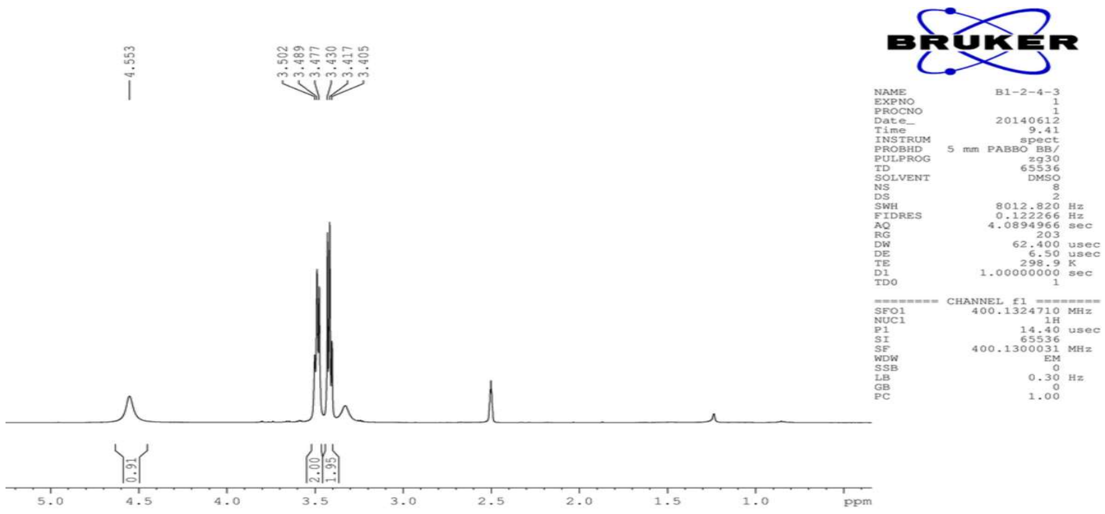

Figure S32. The ^{1}H-NMR (400 MH*z*) spectrum of compound **20** in DMSO-d_6.
